# Improving the Electrochemical Glycerol-to-Glycerate Conversion at Pd Sites via the Interfacial Hydroxyl Immigrated from Ni Sites

**DOI:** 10.3390/molecules29163890

**Published:** 2024-08-16

**Authors:** Yang Zhang, Lin Wang, Shengmin Pan, Lin Zhou, Man Zhang, Yaoyue Yang, Wenbin Cai

**Affiliations:** 1 Key Laboratory of General Chemistry of the National Ethnic Affairs Commission, School of Chemistry and Environment, Southwest Minzu University, Chengdu 610041, China; 220703042004@stu.swun.edu.cn (Y.Z.); wanglin1320310@163.com (L.W.); 202231201091@stu.swun.edu.cn (S.P.); 202231201174@stu.swun.edu.cn (L.Z.); 2 Shanghai Key Laboratory of Molecular Catalysis and Innovative Materials, Collaborative Innovation Center of Chemistry for Energy Materials, Department of Chemistry, Fudan University, Shanghai 200438, China

**Keywords:** glycerol oxidation reaction, glyceric acid/glycerate, Faradaic efficiency, Pd, Ni foam

## Abstract

The electrochemical conversion of glycerol into high-value chemicals through the selective glycerol oxidation reaction (GOR) holds importance in utilizing the surplus platform chemical component of glycerol. Nevertheless, it is still very limited in producing three-carbon chain (C_3_) chemicals, especially glyceric acid/glycerate, through the direct oxidation of its primary hydroxyl group. Herein, Pd microstructure electrodeposited on the Ni foam support (Pd/NF) is designed and fabricated to achieve a highly efficient GOR, exhibiting a superior current density of ca. 120 mA cm^−2^ at 0.8 V vs. reversible hydrogen electrode (RHE), and high selectivity of glycerate at ca. 70%. The Faradaic efficiency of C_3_ chemicals from GOR can still be maintained at ca. 80% after 20 continuous electrolysis runs, and the conversion rate of glycerol can reach 95% after 10-h electrolysis. It is also clarified that the dual-component interfaces constructed by the adjacent Pd and Ni sites are responsible for this highly efficient GOR. Specifically, Ni sites can effectively strengthen the generative capacity of the active adsorbed hydroxyl (OH_ad_) species, which can steadily immigrate to the Pd sites, so that the surface adsorbed glycerol species are quickly oxidized into C_3_ chemicals, rather than breaking the C–C bond of glycerol; thus, neither form the C_2_/C_1_ species. This study may yield fresh perspectives on the electrocatalytic conversion of glycerol into high-value C_3_ chemicals, such as glyceric acid/glycerate.

## 1. Introduction

Biodiesel, as a plentiful and renewable source of hydrocarbons, is an essential ingredient in the search for environmentally friendly energy sources. In biodiesel production, glycerol is a notable by-product (4 million tons [1]) and can serve as the platform chemical [2]. However, glycerol now suffers a serious problem of global oversupply [3,4]. Thus, it is critical to develop efficient methods for high-value application of glycerol resources [5,6]. The electrochemical selective glycerol oxidation reaction (GOR) is an attractive strategy for the value-added conversion of glycerol ($0.11 per kilogram of crude glycerol) into glycerol acid ($1805 per gram) [2]. Glyceric acid is a crucial C_3_ chemical raw material and in high demand for use in insecticides, pharmaceuticals, energy, and other commodity fields [7,8]. However, the presence of triple hydroxyl groups on glycerol poses challenges to the efficiency of GOR and the selectivity of C_3_ chemicals [9]. Therefore, in order to facilitate the intended conversion of glycerate into highly valuable C_3_ compounds, it is imperative to investigate and create extremely effective electrocatalysts.

In the past, the investigation of the GOR to C_3_ chemical process over Pt-based heterogeneous catalysts has received extensive attention [10]. Regarding GOR on Pt catalysts, glycerol is firstly deprotonated and adsorbed on the surface of the electrode, and then the adsorbed surface hydroxyl (OH_ad_) species on the catalyst surface captures the H of the hydroxyl in glycerol to produce an intermediate reaction that further forms C_3_ chemicals [11,12,13]. However, the sluggish kinetics of GOR on Pt is still unresolved, and the use of the noble metal Pt can raise the production costs dramatically [11,14,15,16,17]. As one of the Pt-group metals, Pd has been generally considered to be an alternative to Pt in the oxidation of small organic molecules [18,19,20,21,22], which is also relevant to the GOR process [23,24]. Recently, Holade et al. [25] reported Pd/C for GOR to generate C_3_ chemicals with selectivity of ~30%. Terekhina et al. [26] used Pd_OCTA_ NP catalysts for high GOR activity, achieving a glycerol conversion of 21% at 20 °C. Although Pd catalysts showed high GOR activity, Pd can facilitate the C–C bond cleavage of glycerol during the GOR process due to the intrinsic electron transfer effect, generating C_2_ (glycolate, GA) and C_1_ (formate, FA) chemicals.

Based on the well accepted reaction pathways of GOR [27,28], it is essential and critical to alleviate and even poison the C–C bond-breaking process on the Pd surface, which could lead to the fast and extensive production of C_3_ chemicals. Pd-based composite catalysts were considered as highly active catalysts for GOR due to the increasing capacity to produce active OH_ad_ species. Zalineeva et al. [29] reported self-supported Pd_1_Sn_1_, which was a very active and selective catalyst for C_3_ carboxylate chemicals. The results confirmed that the presence of Sn annihilated the ability of Pd to activate the dissociative adsorption of glycerol with C–C bond breaking and suppressed the formation of carbonate/CO_2_. Meanwhile, the self-supported Pd_4_Bi catalyst was also highly selective toward C_3_ chemicals and enhanced the adsorption of OH_ad_ at sites adjacent to Bi [30]. However, aldehyde and ketone products were dominant. To generate the most highly valued glycerate product among C_3_ chemicals, Mo et al. [31] designed PdCu composite catalysts with tunable bimetallic sites to supply more Pd-OH_ad_ by a laser-assisted nanomaterial preparation method. As a result, PdCu catalysts exhibited high GOR performance with glycerate selectivity of >50%. Holade et al. [25] also confirmed the presence of a large glyceric acid pathway with a selectivity of >30% on PdM/C (M = Ag, Ni) nano-catalysts. Hence, Pd-based composite catalysts, by optimizing the coordination of metal sites, have achieved a highly efficient GOR. These investigations have shown that adjusting the surface structure, especially the local chemical environment of Pd-based catalysts, to increase catalytic activity and selectivity is both feasible and crucial. However, it is still a great challenge to generate high C_3_-chemical selectivity and long-term durability for GOR [32], and there is much scope for improvement. Hence, a self-supported Pd catalyst on an oxyphilic Ni support is designed to achieve a more efficient and durable glycerol-to-C_3_ chemical catalysis. 

In this work, we prepared a Pd overlayer on the nickel foam using a simple one-step electrodeposition method for enhancing GOR performance. As a result, Pd/Ni exhibited excellent GOR activity, stability and high glycerate selectivity. We also clarified the critical role of the abundant OH_ad_ species immigrating from the Ni sites in the elementary steps during GOR needed to produce glycerate. This work is expected to offer guidance for the feasible surface engineering of functionalities to enhance GOR activity and glycerate selectivity.

## 2. Results and Discussion 

### 2.1. Structure of Catalysts

**Figure 1:**
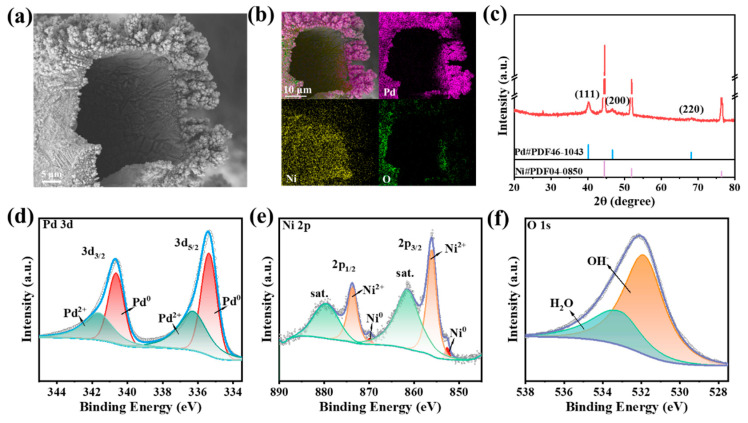
(**a**) Typical SEM image of Pd/NF Catalyst. (**b**) The corresponding EDX mapping images. (**c**) XRD pattern of Pd/NF. (**d**–**f**) Core-level XPS spectra of Pd 3d, Ni 2p, and O 1s orbital of Pd/NF.

Pd overlayer was electrodeposited on the pre-treated Ni foam (NF) surface through a simple potentiostatic strategy. The morphology of the as-obtained Pd/NF catalyst was investigated using a Scanning electron microscope (SEM). Figure 1a shows that Pd was increased at −0.23 V vs. reversible hydrogen electrode (RHE) on NF with vertically aligned seaweed-like structures by uniformly accumulating and outwardly extending. Figure S1 shows homogeneous Pd particles on NF. The size of Pd particles on the Ni foam was investigated at different deposition potentials from −0.23 V vs. RHE to 0.17 V vs. RHE, as shown in Figure S2. Pd particles size of Pd/NF at −0.23 V vs. RHE (152 nm) were smaller than those of Pd/NF at −0.03 V vs. RHE (453 nm) and Pd/NF at 0.17 V vs. RHE (1.5 µm). As the electrodeposition potential increased, the Pd particles’ size gradually become larger, the agglomeration phenomenon even occurring at Pd/NF at 0.17 V vs. RHE. Among them, even granular Pd particles on NF were obtained at −0.23 V vs. RHE. Meanwhile, the corresponding EDX mapping of Pd/NF (Figure 1b) showed the presence of Pd, Ni, and O, as well as the even distribution of Pd and Ni components, suggesting the successful growth of the Pd layer on the surface of the Ni framework (even if this was not by design), which might possess a higher utilization rate for precious metals, given that the GOR was a surface reaction. Note that the structure and chemical state of the Pd/NF samples seems the same for different deposition conditions (Figure S3). Nevertheless, the more preferred seaweed-like structure of the Pd layer could effectively roughen the catalysts surface, resulting in a considerable electrochemical active surface area (ECSA) (vide infra) and thus a better GOR activity. 

Furthermore, the bulk crystalline structure of Pd/NF samples was investigated by X-ray diffraction (XRD). Their diffraction patterns were aligned to those of the reference degrees of standard monoclinic phase Pd (JCPDS 46-1043) and Ni (JCPDS 04-0850) in Figure 1c [33,34]. This indicated that typical but independent face-centered cubic (*fcc*) Pd and Ni crystalline phases formed in the Pd/NF sample, without a Pd–Ni alloy phase or another phase being detectable. 

In addition, the surface composition and chemical states of Pd/NF were investigated via X-ray photoelectron spectroscopy (XPS). The high-resolution Pd 3d XPS spectra (Figure 1d) of Pd/NF exhibited prominent peaks at 340.63 eV (Pd 3d_3/2_) and 335.32 eV (Pd 3d_5/2_), assigned to metallic Pd [35]. The Ni 2p spectra (Figure 1e) exhibited peaks for Ni^0^ and Ni^II^ species at 869.91 eV (Ni 2p_1/2_) and 852.68 eV (Ni 2p_3/2_), and 873.73 eV (Ni 2p_1/2_) and 856.08 eV (Ni 2p_3/2_), respectively [34]. The high proportion of Ni^II^ species at the Pd/NF surface suggests its oxyphilic feature, even when suffering short-term exposure to air before the XPS measurements. This was also supported by the O 1s spectra with the feature peak of 531.84 eV assigned to surface adsorbed oxygen-containing species (OH^−^) [36,37]. Therefore, a highly oxyphilic surface was obtained for the Pd/NF sample although the Pd overlayer and Ni support show weak interaction, which was very positive for the fast oxidation of the C_3_ intermediates of glycerol (see below for details). 

### 2.2. GOR Activity and Selectivity at Pd/NF 

**Figure 2:**
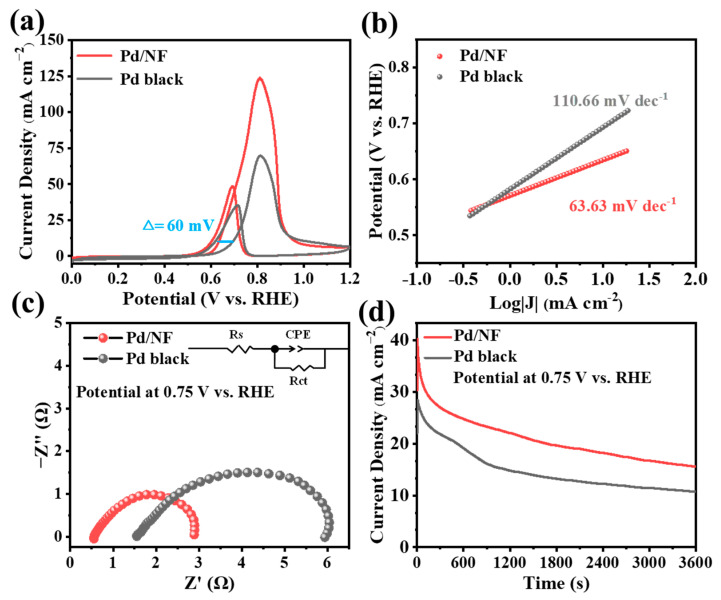
(**a**) CV curves collected on Pd/NF and Pd black electrocatalysts in 1 M NaOH and 0.1 M glycerol solution at a scan rate of 10 mV s^−1^. (**b**) Tafel profiles at Pd/NF and Pd black surface during GOR. (**c**) Electrochemical impedance spectroscopy (EIS) Nyquist plots in 1 M NaOH and 0.1 M glycerol at the potential of 0.75 V vs. RHE. The inset shows the equivalent circuit for EIS measurements. (**d**) Time–current curves for GOR at two catalysts collected at 0.75 V vs. RHE.

The GOR performance at Pd/NF and Pd black was evaluated in an H-cell with typical three-electrode configuration in alkaline media, in which the Pd/NF was used directly as the working electrode without additional binders and substrates. Figure 2a shows the corresponding cyclic voltammograms (CVs). Pd/NF exhibited a high current density of 123 mA cm^−2^ at 0.81 V vs. RHE during GOR, which was 1.8 times as much as Pd black. Figure S4 shows the performance of Pd/NF at different electrodeposition potentials. The current densities of Pd/NF (−0.03 V vs. RHE) and Pd/NF (0.17 V vs. RHE) were 95.1 mA cm^−2^ and 64.1 mA cm^−1^, respectively, indicating that Pd/NF (−0.23 V vs. RHE) had the highest activity for GOR. This may be because the small and even granular particles of Pd/NF (−0.23 V vs. RHE) were beneficial for a good GOR.

In addition, the GOR onset potential at Pd/NF was at ca. 0.60 V vs. RHE, which was far below that at Pd black (0.70 V vs. RHE). The higher GOR apparent activity at the Pd/NF surface should be relative to its high ECSA as estimated by the double-layer capacitance in Figure S5. Characteristic CO-stripping voltammograms of the Pd/NF electrodes are shown in Figure S6. Pd/NF exhibited a high ECSA of 177.4 m^2^ g^−1^_Pd_, suggesting a larger roughness with which to enhance GOR activity. Moreover, the Tafel slope of GOR at Pd/NF was 63.6 mV dec^−1^ in Figure 2b, lower than that at Pd black (110.7 mV dec^−1^). This result suggested that the reaction kinetics for GOR on Pd/NF was faster. Meanwhile, the *R*_ct_ (2.4 Ω) of Pd/NF was smaller than that of Pd black (4.5 Ω) during GOR in Figure 2c, indicating a fast electron transporting process at the Pd/NF–electrolyte interface. These results suggested that Pd/NF was indeed intrinsically more active for GOR than pristine Pd. In addition, the high stability of the Pd/NF catalyst was ensured during GOR in Figure 2d; specifically, 38.8% of the Faradaic current of GOR remained at Pd/NF after a typical 3600-s continuous electrolysis, which is further confirmed by repeated continuous electrolysis, as described below. 

The GOR products at the Pd/NF surface were qualitatively analyzed by high-performance liquid chromatography (HPLC), ^1^H nuclear magnetic resonance (NMR), in situ infrared absorption spectral (IRAS) and ion chromatography (IC). As shown in Figure S7, the reaction residual collected at 0.65–0.75 V vs. RHE can readily detect glycerate, tartronate (TA), lactate (LA), GA, oxalate (OA) and FA products, as compared with the reference HPLC profile obtained at the same experimental conditions. These products were also demonstrated by the ^1^H-NMR (Figure S8) and in situ IRAS results (Figure S9). Meanwhile, the carbonate product was also detected by IC (Figure S10), which was one of the products of GOR at the Pd/NF surface, even though only a trace. 

On the other hand, the interpolation method based on the external standard curves estimated by HPLC were used to proceed with quantitative analysis of GOR products. The HPLC profiles of the above-mentioned eight typical chemicals with different concentrations and their corresponding fitted external standard curves were obtained and shown in Figures S11–S15. Thus, the selectivity and Faradaic efficiency of various GOR products at Pd/NF could be readily evaluated according to the equations described in Section 3. Error bars were produced for the FE of the products, especially for the glycerate, which had a small error of <1.5%. The error of other products was about 5%.

**Figure 3:**
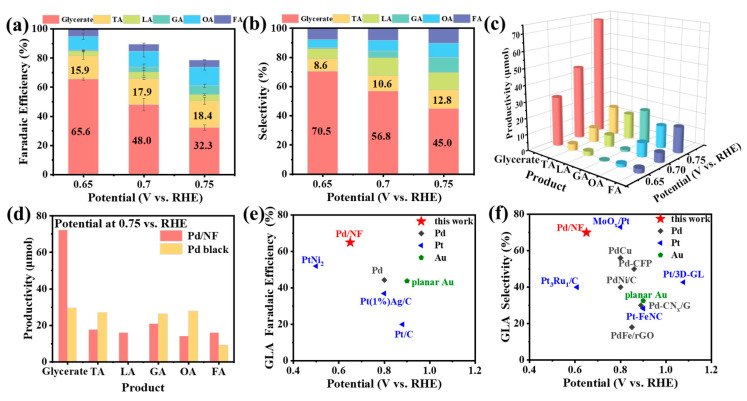
The histogram of Faraday efficiency (*FE*) (**a**) and selectivity (**b**) of GOR products electrolyzed in 1 M NaOH with 0.1 M glycerol on Pd/NF. (**c**) Productivity on Pd/NF in 1 M NaOH with 0.1 M glycerol after 1-h electrolysis. (**d**) Productivity of all products on Pd/NF and Pd black after 1-h electrolysis at 0.75 V vs. RHE. (**e**,**f**) A brief comparison of *FE* and selectivity different catalysts (Refs. [14,15,16,25,31,33,36,38,39,40,41,42,43] in the manuscript).

As shown in Figure 3a,b, the apparent *FE* and selectivity of C_3_ chemicals was more than 80% at 0.65 V vs. RHE, indicating that the reaction pathway of C_3_ chemicals dominated at the Pd/NF surface. In particular, the *FE* of glycerate was as high as 65.6%, associated with its selectivity and productivity reaching 70.5% and 72.4 µmol h^−1^ (Figure 3d), respectively. With the increase of the applied potential from 0.65 V vs. RHE to 0.75 V vs. RHE, glycerate was still the main product, with a productivity of ≥72.4 µmol h^−1^, as seen in Figure 3c. In contrast, the selectivity of C_3_ chemicals on Pd black was only 47.2% at 0.75 V vs. RHE, as shown in Figure S16, consisting of glycerate (24.7%), TA (22.5%), GA (21.9%), OA (24.7%) and FA (7.6%), respectively. Compared with Pd/NF, GOR at the pristine Pd surface were more likely to proceed to deep oxidation to generate TA or C_2_ chemicals. In addition, Figure 3d directly exhibits that glycerate productivity of Pd/NF (72.4 µmol h^−1^) was ~2.45 fold higher than that of Pd black (29.6 µmol h^−1^), which was higher than that of Pd/NF@−0.03 V vs. RHE (60.0 µmol h^−1^) and Pd/NF@0.17 V vs. RHE (37.7 µmol h^−1^) in Figures S17 and S18. This may be due to the fact that the Pd/NF (−0.23 V vs. RHE) had a smaller Pd particle size, which led to easier adsorption of glycerol and accelerated the rate of the reaction to generate glycerate.

Furthermore, the apparent *FE* and selectivity of GOR were compared in recently published work. As shown in Figure 3e, *FE* of glycerate at Pd/NF was 65.6% at low potential of 0.65 V vs. RHE, being obviously superior to other Pt or Au catalysts [14,15,31,38,39]. Meanwhile, the selectivity of glycerate (>70.5%) was beyond that of most Pt-based catalysts, as shown in Figure 3f. Therefore, it can be concluded that the Pd/NF exhibits high active catalytic performance for GOR to obtain C_3_ chemicals compared with the reported works [16,25,31,33,36,38,40,41,42,43], especially for glycerate.

### 2.3. GOR Stability at Pd/NF

**Figure 4:**
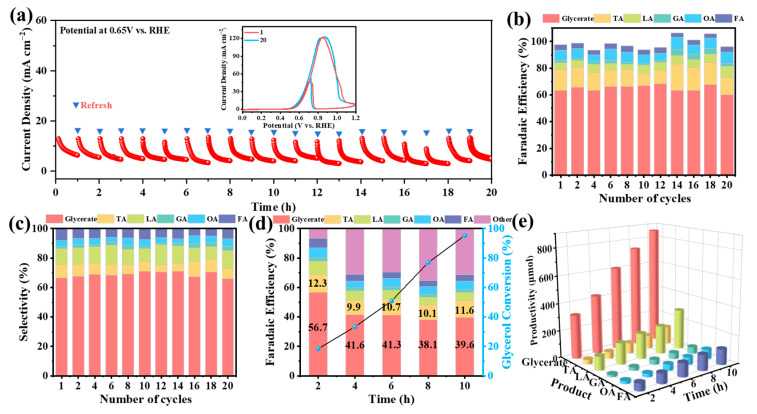
(**a**) The *i*-t profiles of multiple 1-h electrolysis of glycerol oxidation at Pd/NF in 1 M NaOH and 0.1 M glycerol, controlled at 0.65 V vs. RHE. The inset shows the CV curves taken in 1 M NaOH and 0.1 M glycerol before the electrolysis and after 20 cycles of continuous GOR. (**b**,**c**) *FE* and selectivity of GOR products after 20-h electrolysis runs at Pd/NF. (**d**) *FE* and glycerol conversion at Pd/NF at different electrolysis times at 0.75 V vs. RHE. (**e**) Productivity of various products after electrolyzing for different times on Pd/NF surface.

Long-term stability has been a crucial indicator for electrosynthesis and the activity of Pd-based electrocatalysts usually showed a rapid decline in most reports [35]. Therefore, the GOR durability measurements by multiple anodic electrolysis at a constant potential of 0.65 V vs. RHE were performed on the as-obtained Pd/NF catalysts. As shown in Figure 4a, after 20 continuous runs at the Pd/NF surface, the GOR Faradaic current density was still maintained at ca. 120 mA cm^−2^, indicating negligible changes to Pd/NF during GOR after 20-h electrolysis. Meanwhile, the Pd/NF samples were seldom found to occur at the surface and/or in bulk reconstruction, even if they were subjected to rigorous electrochemical testing. This can be proved by almost the same XRD pattern (Figure S19) and XPS spectra (Figure S20) collected before and after the long-term electrolysis. 

On the other hand, the dominated GOR products after electrooxidation were still C_3_ chemicals (apparent *FE* and selectivity > 80%). Among them, the apparent *FE* of glycerate was ca. 65% with selectivity of ca. 70% in Figure 4b,c. Nevertheless, it has to be admitted that deep oxidation undeniably occurred when one single electrolysis lasted for over 4 h (Figure 4d). The apparent *FE* of all C_3_ chemicals dropped from ca. 80% (1–2 h) to ca. 50% (>4 h), which means glycerate can be further electrooxidized. Given that CO_3_^2−^ can be detected by IC after 4-h electrolysis, here we speculated that the *FE* contribution from other products in Figure 4d could be the deep oxidation of C_3_ and/or C_2_ intermediates to CO_2_/carbonate. Fortunately, the conversion rate of glycerol was as high as 95% (accompanied with a superior glycerate yield of 895.93 µmol) after 10-h electrolysis, shown in Figure 4e, and greater than recent reports [25,44]. 

### 2.4. The Promoting Effect of Pd/NF on GOR

**Figure 5:**
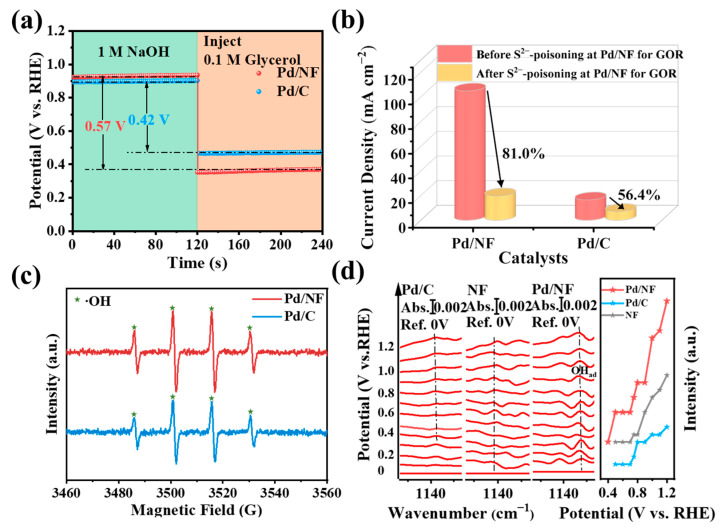
(**a**) Time-dependent OCP profiles collected at Pd/NF and Pd/C in 1 M NaOH solution without and with 0.1 M glycerol. (**b**) Comparison of peak current densities for GOR in 1 M NaOH solution with 0.1 M glycerol on Pd/NF and Pd/C before and after their immersion in 3.8 mM Na_2_S solution. (**c**) EPR spectra of the residual electrolytes after the Pd/NF and Pd/C working electrode were applied with a constant potential of 0.65 V vs. RHE in 1 M NaOH with 0.1 M glycerol for 300 s using DMPO as the trapping agent. (**d**) In situ ATR-IR spectra of Pd/C, NF and Pd/NF in Ar-saturated 0.1 M NaOH with potential scanning from 0 to 1.2 V. The right panel is the potential-dependent intensity variation of the IR band of OH_ad_ species at the above three electrode surfaces.

It was clear that Pd/NF showed superior glycerol-to-glycerate electrocatalysis, but the promoting effect should be further probed. Initially, open circuit potential (OCP) variation of Pd/NF and Pd/C induced by glycerol was conducted and compared to evaluate glycerol adsorption (Figure 5a). After the injection of 0.1 M glycerol, the OCP at Pd/NF decreased by 0.57 V vs. RHE, being higher than that of pristine Pd (0.42 V vs. RHE). This result indicated a strong glycerol adsorption on Pd/NF and could be helpful for the further oxidation of glycerol. 

Furthermore, S^2−^ ions with strong adsorption were introduced to the Pd/NF and Pd/C catalysts’ surface to indirectly clarify the real catalytic active sites. The CVs were collected in 1 M NaOH solution with and without glycerol after immersing Pd/NF and Pd/C in Na_2_S solution for 1 h to poison the surface active sites. As shown in Figure S21a,b, the hydrogen adsorption/desorption and the Pd/Pd-OH_ad_ redox pairs all disappeared as expected, indicating the strong poisoning effect of S^2−^ on surface Pd sites. Note that S^2−^ ions can also stably dwell at Ni sites. On this premise, it was not strange that the decrease in GOR current density is very different in the two conditions. Namely, the current density of GOR at the S^2−^-adsorbed Pd/NF surface decreased by 81.0% (Figure S21c), being much greater than that at the S^2−^-adsorbed pristine Pd surface (56.4% in Figure S21d) in Figure 5b. This result indirectly suggested that Ni support could also contribute to the GOR process rather than only serving as a support, and the interfacial domains formed by the adjacent Pd and Ni sites might be the real catalytic active sites for GOR. Given the oxyphilic feature of Ni metals, here we reasoned that Ni sites could be a supplier of OH_ad_ species, which should be the key species for oxidizing the surface carbonaceous species from glycerol, according to the Langmuir–Hinshelwood mechanism [45]. The Bode Phase plots were collected at the Pd/NF surface with and without glycerol at around 10 Hz, exhibiting an obvious high-frequency and low-phase shifting, suggesting the fast consumption of the surface OH_ad_ species at the voltametric peak of glycerol oxidation at 0.6–0.8 V vs. RHE, as shown in Figure S22 [46]. This result can emphasize the key role of OH_ad_ species in glycerol oxidation (vide infra). 

To further prove this speculation, electron paramagnetic resonance (EPR) at Pd and Pd/NF in NaOH and in situ attenuated total-reflection infrared adsorption spectra (ATR-IR), during positive voltammetry scanning in 1 M NaOH, were carried out. EPR experiments were employed to identify active OH˙ radicals using 5,5-dimethyl-1-pyrrole oxide (DMPO) as the trapping agent. A strong characteristic peak of DMPO-OH˙ substance was found on Pd/NF surface (Figure 5c and Figure S23), indicating the form of more OH˙ radicals on the surface of Pd/NF due to the introduction of Ni sites with an oxyphilic role [35,47]. It could be predictable that the higher concentration of OH˙ radicals in the double-layer, the more possibility that OH_ad_ species can be obtained at the catalyst’s surface. Subsequently, the adsorption and coverage of active OH_ad_ species on the surface of Pd/NF was explored by in situ ATR-IR measurements (Figure 5d). The band at ca. 1120–1140 cm^−1^ was assigned to the O–H bending vibration of OH_ad_ species at Pd and Ni sites [48,49,50]. The band intensities of OH_ad_ species at Pd/C and NF were found to be rather weak; in contrast, much higher levels of OH_ad_ species were shown at a low potential (0.4 V vs. RHE) on the surfaces of Pd/NF. This means that high-coverage OH_ad_ species were formed at the Pd–Ni domain, which should be propitious for the fast oxidation of glycerol to glycerate rather than breaking its C–C bond to form C_2_/C_1_ products. Meanwhile, it can also suggest that Pd sites of Pd/NF could borrow OH_ad_ species formed at Ni sites; in other words, OH_ad_ species could immigrate from Ni sites to the neighboring Pd sites, which is in agreement with the “mean-field approximation” kinetics in the framework of the Langmuir–Hinshelwood mechanism [45,51,52,53].

**Figure 6:**
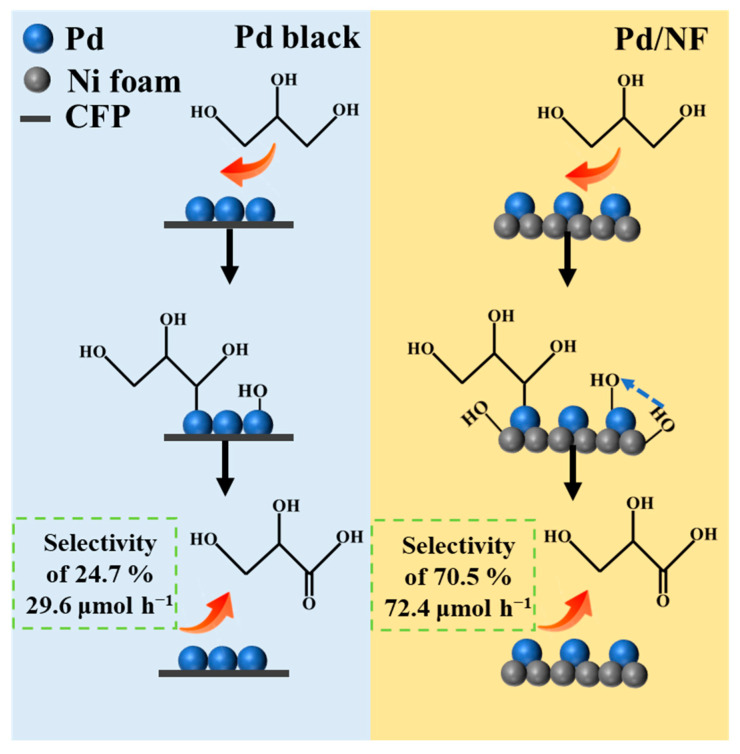
The scheme for the selective oxidation of glycerol into glycerate at Pd/NF and Pd black surfaces, which emphasizes the pivotal role of the population of surface OH_ad_ species in the direct conversion from glycerol to glycerate.

Therefore, Pd/NF enhanced the oxidation from glycerol to C_3_ chemicals by providing more OH_ad_ species with the help of Ni sites, where the immigration of OH_ad_ species from Ni to Pd sites should be pivotal for the implementation. Consequently, the reaction pairs of C_3_ intermediates and OH_ad_ species to generate the chemicals consist of the glycerate (70.5%) at the potential of 0.65 V vs. RHE (Figure 6). According to the kinetics model of parallel reaction, this actually alleviates the cleavage of the C–C bond of glycerol and thus generates more C_3_ chemicals, rather than producing a great proportion of GA and FA. as happens at the pristine Pd surface (Figure S16).

## 3. Experimental Section

### 3.1. Chemicals and Materials

Palladium(II) chloride (PdCl_2_, 7647-10-1, ≥99.00%), hydrochloric acid (HCl, 7647-01-0, ≥36.00%), sulfuric acid (H_2_SO_4_, 7664-93-9, ≥95.00%), sodium chloride (NaCl, 7647-14-5, ≥99.50%), glycerol (C_3_H_8_O_3_, 56-81-5, ≥98.00%), sodium hydroxide (NaOH, 1310-73-2, ≥98.00%), sodium borohydride (NaBH_4_, 1310-73-2, ≥99.8%), sodium Carbonate Anhydrous (Na_2_CO_3_, 497-19-8, ≥99.8%), formic acid (HCOOH, 64-18-6, ≥98.00%), and oxalic acid dihydrate (C_2_H_2_O_4_·2H_2_O, 6153-56-6, ≥99.50%) were purchased from Chengdu Cologne Chemical Co., Ltd. (Chengdu, China). DL-glyceraldehyde (C_3_H_6_O_3_, 80-69-3, ≥90.00%), 2,3-dihydroxypropanoic acid (20% in water) (C_3_H_6_O_4_, 473-81-4, ≥95.00%), tartronic acid (C_3_H_4_O_5_, 80-69-3, ≥99.8%), 1,3-dihydroxyacetone (C_3_H_6_O_3_, 96-26-4, ≥99.00%), lactic acid (C_3_H_6_O_3_, 50-215, ≥85.00%), and glycolic acid (C_2_H_4_O_3_, 79-14-1, ≥99.00%) were purchased from Chengdu Shu Test Co., Ltd. (Chengdu, China). Active carbon powder (Vulcan-XC72) was purchased from Shanghai Cabot Chemical Co., Ltd. (Chengdu, China). Nafion solution (Dupont) was purchased from Suzhou Sinero Technology Co., Ltd. (Suzhou, China). Nickel foam was purchased from Jiayisheng foam Metal Co., Ltd. (Kunshan, China). Specifications for other chemicals and reagents are as follows: deionized water (Milli-Q, 18.25 MΩ cm), high-purity Ar (99.999%), high-purity N_2_ (99.999%). All chemical reagents were used exactly as they were received with no further purification.

### 3.2. Synthesis of Catalysts

#### 3.2.1. Synthesis of Pd/NF Catalysts

NF was used as matrix for growing the Pd overlayer. Firstly, a piece of NF (10 × 20 × 1.5 mm) was cleaned ultrasonically in 1 M HCl (50 mL) for 10 min, anhydrous ethanol (50 mL) for 10 min, deionized water (50 mL) and anhydrous ethanol (50 mL) for 10 min, respectively. Then, the treated NF was dried under room temperature. After that, the Pd overlayer on NF was prepared by the simple electrodeposition method, denoted as Pd/NF [54]. The electrolyte consisted of 3 mM PdCl_2_, 200 mM H_3_BO_3_, and 200 mM NaCl. The electrodeposition process was carried out in a three-electrode system with the treated NF as the working electrode, the saturated Ag/AgCl (saturated potassium chloride solution) electrode as the reference electrode, and the Pt foil as the counter electrode under a potential of −0.23 V vs. RHE. The electrode was dried in a vacuum oven at 50 °C to obtain the Pd/NF catalysts.

#### 3.2.2. Synthesis of Pd/C and Pd Black Catalysts

The Pd/C catalysts were synthesized by the liquid phase reduction method. A total of 3.97 mL of 11.84 mM PdCl_2_ solution, 10 mL deionized water and 20 mg of XC-72R carbon powder were mixed in a clean beaker using ultrasonic, denoted as solution A. A total of 71.1 mg Na_2_CO_3_ and 35.5 mg NaBH_4_ were dissolved in 5 mL deionized water, denoted as solution B. Subsequently, solution B was slowly dripped into solution A. The mixture was stirred in an ice-water bath overnight and then washed by deionized water. The sample was dried in a vacuum oven at 60 °C to obtain Pd/C catalysts. Pd black catalysts were prepared using the same method expect that XC-72R carbon powder was absent.

### 3.3. Characterization

SEM images were recorded by a Zeiss Gemini SEM 360 (Carl Zeiss AG, Oberkochen, Germany, 2 kV). XRD patterns were collected on a XPert Pro MPD diffractometer using a Cu Kα source, with a scan range of 10–80° and scan step of 1 degree min^−1^. XPS were performed on a Thermo Fisher Scientific (Waltham, MA, USA) K-Alpha X-ray photoelectron spectrometer using Al Kα X-rays as the excitation source (*hv* = 1486.68 eV). Metal contents in catalysts were determined by inductively coupled plasma optical emission spectrometer (ICP-OES) on a Thermo Fisher iCAP 7400. EPR measurements were carried out using a BRUKE EMX (Bruker Company in Billerica, MA, USA) at room temperature. The magnetic parameters of the radicals detected were obtained using the magnetic field and the microwave frequency.

### 3.4. Electrochemical Measurements

The activity of glycerol oxidation reaction was tested by CV at a scan rate of 10 mV s^−1^ in a high-purity Ar-saturated 1 M NaOH and 0.1 M glycerol solution using Pd/NF, saturated calomel electrode and a carbon rod as the working electrode, reference electrode and counter electrode, respectively. In addition, the loading mass with 2.04 mg cm^−2^ of Pd on NF was confirmed using ICP-OES in Table S1. The stability of the catalysts was also tested by chronoamperometry. Electrochemical impedance spectroscopy (EIS) was measured from 0.01 mHz to 100 kHz at 0–1.2 V vs. RHE with an amplitude of 5 mV. In order to conduct a controlled experiment, the powder catalyst (Pd black) was dispersed in ethanol aqueous solution with Nafion by ultrasonic treatment and sprayed onto carbon fiber paper (CFP) with a mass load of 2 mg cm^−2^. The testing method was consistent with the above. The powder catalyst Pd/C was loaded on the surface of the polished glassy carbon electrode. The scan rate of cyclic voltammetry tests was 50 mV s^−1^. Potential control and current recording were performed on a CHI660E electrochemical workstation. All electrolytes were freshly prepared from guaranteed reagent and ultrapure Milli-Q water (18.25 MΩ cm), and all tests were performed at room temperature. All potentials versus saturated calomel electrode (SCE) were transformed into RHE scales using the following Equation (1):

(1) $$E\left(vs.RHE\right)=E\left(vs.SCE\right)+0.242V+0.059\times pH$$

### 3.5. Product Analysis

The chronoamperometry testing at varied potentials was conducted to determine the products of alcohol oxidation. This was first performed using the high-performance liquid chromatography (HPLC, LC-5090 system, FuLi Instruments Co., Ltd. in Taizhou, China) equipped with a sugar column (ChromCore Sugar-10H 7.8 × 300 mm Shimpark GWS 6 µm) and an ultraviolet-visible (UV-Vis) detector (210 nm). The flow rate of the eluent (5 mM H_2_SO_4_) was 0.4 mL min^−1^. 20 µL of electrolyte (electrolyte was neutralized using 0.5 M H_2_SO_4_, and then was diluted by 20 times) was injected into the column under the temperature of the column at 50 °C. Since carbonate ions were not detected by HPLC, they were identified by ion chromatography (IC, Thermo Fisher ICS1600 was purchased from Thermo Fisher Scientific from, Waltham, MA, USA) equipped with a sugar column (Dionex^TM^ IonPac^TM^ AG11-H RFIC^TM^ 4 × 50 mm) and a conductivity detector. The flow rate of the eluent (20 mM KOH) was 1 mL min^−1^. 20 µL of electrolyte (electrolyte was diluted by 20 times with deionized water) was injected into the column under the temperature of the column at 35 °C. In addition, the products were also confirmed by the NMR spectrometer (Bruker AVANCE NEO 600 MHz was purchased from Bruke Company in Billerica, MA, USA).

The *FE* of products was calculated based on their corresponding electron transfer per molecule oxidation using the following equation:

(2) $$FE=\frac{n_{e}\times n_{products}\times F}{Q_{total}}\times 100\%$$

where *n_e_* is the number of electrons required to oxidize glycerol to products. *n_products_* is the productivity of products, *F* is Faraday constant (*F* = 96,485 C mol^−1^), and *Q_total_* is the quantity of electric charge.

The selectivity of products was calculated based on total moles of glycerol oxidation products using the following equation:

(3) $$Selectivity=\frac{n_{product}}{n_{total}}\times 100\%$$

Calculate the conversion rate of glycerol using the following equation:

(4) $$X_{glycerol}=\frac{n_{glycerol,in}-n_{glycerol,rem}}{n_{glycerol,in}}\times 100\%$$

where *n*_*glycerol*,*in*_ is the initial molar amount of glycerol, *n*_*glycerol*,*rem*_ is the remaining molar amount of glycerol.

### 3.6. In Situ Electrochemical Infrared Spectral Measurements

The real-time electrochemical ATR-IR measurements of the catalysts were described in previous work elsewhere [48,55,56,57]. The in situ ATR-IR experimental procedure involved the chemical deposition of an approximately 60 nm thick Au island-like nanofilm on the reflection plane of a cylindrical single-crystal Si prism. Next, the Pd/NF and blank NF (10 × 10 × 1.5 mm) were glued onto the Au surface using a 1 mL ethanol aqueous solution containing 120 μL of 5 wt.% Nafion. As contrast, the catalyst (Pd/C) ink was pipetted onto the Au surface with the Pd loading mass of ca. 45 mg cm^−2^. After drying, the as-obtained electrode was used as WE in the subsequent electrochemical in situ ATR-IR measurements, employing carbon paper as the counter electrode and SCE as the reference electrode, respectively. The homemade spectro-electrochemical cell was filled with 30 mL of Ar saturated 0.1 M NaOH solution with or without 0.1 M glycerol. The in situ ATR-IR spectra were collected every 2 s during the positive linear voltametric scanning with a sweeping rate of 2 mV s^−1^.

The IRAS measurements were similar to the procedures in previous work [56,58,59]. Specifically, the catalyst on NF was scraped off and configured into ink with a 1 mL aqueous ethanol solution containing 120 µL 5 wt.% Nafion, denoted as Pd/NF ink. The ink for the Pd/C catalyst was also prepared by the same protocol, denoted as Pd/C ink. Next, 8 µL of the aforementioned ink was dipped onto a smooth glass carbon electrode with a 6-mm diameter to act as WE. The SCE served as the reference electrode and the Pt film as the counter electrode. Prior to collecting the spectra, the WE was pressed onto the CaF_2_ prism surface to form a roughly 10 µm thin layer with a 0.1 M glycerol electrolyte and 1 M NaOH. Via the CaF_2_ prism and the solution layer, the infrared beam can travel at an incident angle of 55 degrees, reflect on the WE surface, and ultimately return to the detector. IR spectra were obtained every 5 s using a linear potential scanner operating at 5 mV s^−1^ in 1 M NaOH and 0.1 M glycerol.

The electrode potential was adjusted and the current was recorded using a CHI660E electrochemical workstation. Every potential was changed to match the respective RHE values. In situ ATR-IR measurements were conducted using an Agilent Cary 660 FTIR spectrometer (Agilent Technologies (China), Inc., Beijing, China) fitted with an MCT detector. All the spectra in this work are shown in absorbance units defined as ***A* = −log(*I*/*I*_0_)**, where *I* and *I*_0_ represent the absorption intensities at the sample and correlative conditions, respectively. The spectral resolution was 8 cm^−1^.

## 4. Conclusions

In summary, Pd nanoparticles onto nickel foam were successfully synthesized using a simple one-step electrodeposition method for enhancing GOR performance. As a result, the Pd/NF exhibited excellent GOR activity due to the immigration of Ni with interfacial OH_ad_. The promoting effects of Ni support on the Pd sites have been confirmed by in situ IRAS, EPR, toxicity experiment. Our work clarifies the critical role of Ni sites in enhancing the productivity of interfacial OH_ad_, leading to the high activity, long-term stability and considerable selectivity of glycerate chemicals. Active OH_ad_ and Pd-OH_ad_ bond strength are the elementary steps during GOR to produce glycerate. This work offers a straightforward and effective method to keep noble-based electrocatalysts from deactivating and to selectively extract high-value C_3_ chemicals from glycerol. Nevertheless, the enhancement mechanism of selective oxidation of primary hydroxyl group of glycerol should be further studied for clarification.

## Data Availability

The original contributions presented in the study are included in the article, further inquiries can be directed to the corresponding author.

## Supplementary Materials

The following supporting information can be downloaded at: https://www.mdpi.com/article/10.3390/molecules29163890/s1.

## References

[1] Attarbachi T., Kingsley M.D., Spallina V. (2023). New Trends on Crude Glycerol Purification: A Review. Fuel.

[2] Rahim S.A.N.M., Lee C.S., Abnisa F., Aroua M.K., Daud W.A.W., Cognet P., Pérès Y. (2020). A Review of Recent Developments on Kinetics Parameters for Glycerol Electrochemical Conversion—A By-Product of Biodiesel. Sci. Total Environ..

[3] Liu C.J., Wang H.M., Karim A.M., Sun J.M., Wang Y. (2014). Catalytic Fast Pyrolysis of Lignocellulosic Biomass. Chem. Soc. Rev..

[4] Verma S., Lu S., Kenis P.J.A. (2019). Co-Electrolysis of CO_2_ and Glycerol as a Pathway to Carbon Chemicals with Improved Technoeconomics due to Low Electricity Consumption. Nat. Energy.

[5] Akhade S.A., Singh N., Gutiérrez O.Y., Lopez-Ruiz J., Wang H., Holladay J.D., Liu Y., Karkamkar A., Weber R.S., Padmaperuma A.B. (2020). Electrocatalytic Hydrogenation of Biomass-Derived Organics: A Review. Chem. Rev..

[6] Bricotte L., Chougrani K., Alard V., Ladmiral V., Caillol S. (2023). Dihydroxyacetone: A User Guide for a Challenging Bio-Based Synthon. Molecules.

[7] Yu H.-Z., Hu M.-Y., Chen C., Hu C.-J., Li Q.-H., Hu F., Peng S.-J., Ma J. (2023). Ambient γ-Rays-Mediated Noble-Metal Deposition on Defect-Rich Manganese Oxide for Glycerol-Assisted H_2_ Evolution at Industrial-Level Current Density. Angew. Chem. Int. Ed..

[8] Wang Y., Zhang M., Liu Y., Zheng Z., Liu B., Chen M., Guan G., Yan K. (2023). Recent Advances on Transition-Metal-Based Layered Double Hydroxides Nanosheets for Electrocatalytic Energy Conversion. Adv. Sci..

[9] Chen Z., Liu C., Zhao X., Yan H., Li J., Lyu P., Du Y., Xi S., Chi K., Chi X. (2019). Promoted Glycerol Oxidation Reaction in an Interface-Confined Hierarchically Structured Catalyst. Adv. Mater..

[10] Kwon Y., Schouten K.J.P., Koper M.T.M. (2011). Mechanism of the Catalytic Oxidation of Glycerol on Polycrystalline Gold and Platinum Electrodes. ChemCatChem.

[11] Li Y., Wei X., Pan R., Wang Y., Luo J.-J., Li L.-X., Chen L.-S., Shi J.-L. (2024). PtAu Alloying-Modulated Hydroxyl and Substrate Adsorption for Glycerol Electrooxidation to C_3_ Products. Energy Environ. Sci..

[12] Garcia A.C., Kolb M.J., van Nierop y Sanchez C., Vos J., Birdja Y.Y., Kwon Y., Tremiliosi-Filho G., Koper M.T.M. (2016). Strong Impact of Platinum Surface Structure on Primary and Secondary Alcohol Oxidation during Electro-Oxidation of Glycerol. ACS Catal..

[13] Hu X.-Y., Lu J., Liu Y., Chen L., Zhang X.-W., Wang H.-T. (2023). Sustainable Catalytic Oxidation of Glycerol: A Review. Environ. Chem. Lett..

[14] Luo H., Yukuhiro V.Y., Fernández P.S., Feng J., Thompson P., Rao R.R., Cai R., Favero S., Haigh S.J., Durrant J.R. (2022). Role of Ni in PtNi Bimetallic Electrocatalysts for Hydrogen and Value-Added Chemicals Coproduction via Glycerol Electrooxidation. ACS Catal..

[15] Lima C.C., Rodrigues M.V.F., Neto A.F.M., Zanata C.R., Pires C.T.G.V.M.T., Costa L.S., Solla-Gullón J., Fernández P.S. (2020). Highly Active Ag/C Nanoparticles Containing Ultra-Low Quantities of Sub-Surface Pt for the Electrooxidation of Glycerol in Alkaline Media. Appl. Catal. B Environ..

[16] Li J.-F., Jiang K.-H., Bai S.-H., Guan C.-H., Wei H., Chu H.-B. (2022). High Productivity of Tartronate from Electrocatalytic Oxidation of High Concentration Glycerol through Facilitating the Intermediate Conversion. Appl. Catal. B Environ..

[17] Meng Q.-C., Jin L.-B., Ma M.-Z., Gao X.-Q., Chen A.-B., Zhou D.-J., Sun X.-M. (2023). Highly Dispersed Pt Nanoparticles Root in Single-Atom Fe Sites in LDHs toward Efficient Methanol Oxidation. J. Electrochem..

[18] Shi K., Si D., Teng X., Chen L.-S., Shi J.-L. (2024). Pd/NiMoO_4_/NF Electrocatalysts for the Efficient and Ultra-Stable Synthesis and Electrolyte-Assisted Extraction of Glycolate. Nat. Commun..

[19] Huang X., Feng J., Hu S.-N., Xu B.-Y., Hao M.-S., Liu X.-Z., Wen Y., Su D., Ji Y.-J., Li Y.-Y. (2024). Regioselective Epitaxial Growth of Metallic Heterostructures. Nat. Nanotechnol..

[20] Zhang X.-T., Hui L., Yan D.-X., Li J.-Z., Chen X., Wu H., Li Y.-L. (2023). Defect Rich Structure Activated 3D Palladium Catalyst for Methanol Oxidation Reaction. Angew. Chem. Int. Ed..

[21] Wu W.-X., Wang Y. (2023). Electrochemical Oxidation of Ethylene on Palladium Electrode. J. Electrochem..

[22] Zhang J.-M., Zhang X.-J., Chen Y., Fan Y.-J., Fan Y.-J., Jia J.-F. (2023). Deep Euteceic Solvents-Assisted Synthesis of Novel Network Nanostructures for Accelerating Formic Acid Electrooxidation. J. Electrochem..

[23] Zhou X.-C., Ma Y.-B., Ge Y., Zhu S.-Q., Cui Y., Chen B., Liao L., Yun Q., He Z., Long H. (2022). Preparation of Au@Pd Core–Shell Nanorods with fcc-2H-fcc Heterophase for Highly Efficient Electrocatalytic Alcohol Oxidation. J. Am. Chem. Soc..

[24] Huang B., Ge Y., Zhang A., Zhu S., Chen B., Li G., Yun Q., Huang Z., Shi Z., Zhou X. (2023). Seeded Synthesis of Hollow PdSn Intermetallic Nanomaterials for Highly Efficient Electrocatalytic Glycerol Oxidation. Adv. Mater..

[25] Holade Y., Morais C., Servat K., Napporn T.W., Kokoh K.B. (2013). Toward the Electrochemical Valorization of Glycerol: Fourier Transform Infrared Spectroscopic and Chromatographic Studies. ACS Catal..

[26] He Z.-Y., Hwang J., Gong Z.-H., Zhou M.-Z., Zhang N., Kang X.-W., Han J.W., Chen Y. (2022). Promoting Biomass Electrooxidation via Modulating Proton and Oxygen Anion Deintercalation in Hydroxide. Nat. Commun..

[27] Li T., Harrington D.A. (2021). An Overview of Glycerol Electrooxidation Mechanisms on Pt, Pd and Au. ChemSusChem.

[28] White J., Terekhina I., Campos dos Santos E., Martín-Yerga D., Pettersson L.G.M., Johnsson M., Cornell A. (2024). Synergistic Bimetallic PdNi Nanoparticles: Enhancing Glycerol Electrooxidation While Preserving C3 Product Selectivity. ACS Appl. Energy Mater..

[29] Zalineeva A., Serov A., Padilla M., Martinez U., Artyushkova K., Baranton S., Coutanceau C., Atanassov P.B. (2015). Glycerol Electrooxidation on Self-Supported Pd_1_Sn_x_ Nanoparticules. Appl. Catal. B Environ..

[30] Zalineeva A., Serov A., Padilla M., Martinez U., Artyushkova K., Baranton S., Coutanceau C., Atanassov P.B. (2014). Self-Supported Pd_x_Bi Catalysts for the Electrooxidation of Glycerol in Alkaline Media. J. Am. Chem. Soc..

[31] Mo X., Gao X., Gillado A.V., Chen H.-Y., Chen Y., Guo Z., Wu H.-L., Tse E.C.M. (2022). Direct 3D Printing of Binder-Free Bimetallic Nanomaterials as Integrated Electrodes for Glycerol Oxidation with High Selectivity for Valuable C_3_ Products. ACS Nano.

[32] Goetz M.K., Usman E., Choi K.-S. (2023). Understanding and Suppressing C–C Cleavage during Glycerol Oxidation for C3 Chemical Production. ACS Catal..

[33] Terekhina I., White J., Cornell A., Johnsson M. (2023). Electrocatalytic Oxidation of Glycerol to Value-Added Compounds on Pd Nanocrystals. ACS Appl. Nano Mater..

[34] Si D., Xiong B.-Y., Chen L.-S., Shi J.-L. (2021). Highly Selective and Efficient Electrocatalytic Synthesis of Glycolic Acid in Coupling with Hydrogen Evolution. Chem Catal..

[35] Liu F., Gao X., Shi R., Guo Z., Tse E.C.M., Chen Y. (2023). Concerted and Selective Electrooxidation of Polyethylene-Terephthalate-Derived Alcohol to Glycolic Acid at an Industry-Level Current Density over a Pd−Ni(OH)_2_ Catalyst. Angew. Chem. Int. Ed..

[36] Yu X., dos Santos E.C., White J., Salazar-Alvarez G., Pettersson L.G.M., Cornell A., Johnsson M. (2021). Electrocatalytic Glycerol Oxidation with Concurrent Hydrogen Evolution Utilizing an Efficient MoO_x_/Pt Catalyst. Small.

[37] Xia Z., Ma C., Fan Y., Lu Y., Huang Y.-C., Pan Y., Wu Y., Luo Q., He Y., Dong C.-L. (2024). Vacancy Optimized Coordination on Nickel Oxide for Selective Electrocatalytic Oxidation of Glycerol. ACS Catal..

[38] Kim D., Oh L.S., Tan Y.C., Song H., Kim H.J., Oh J. (2021). Enhancing Glycerol Conversion and Selectivity toward Glycolic Acid via Precise Nanostructuring of Electrocatalysts. ACS Catal..

[39] Yadegari H., Ozden A., Alkayyali T., Soni V., Thevenon A., Rosas-Hernández A., Agapie T., Peters J.C., Sargent E.H., Sinton D. (2021). Glycerol Oxidation Pairs with Carbon Monoxide Reduction for Low-Voltage Generation of C_2_ and C_3_ Product Streams. ACS Energy Lett..

[40] Lee D., Kim Y., Kwon Y., Lee J., Kim T.-W., Noh Y., Kim W.B., Seo M.H., Kim K., Kim H.J. (2019). Boosting the Electrocatalytic Glycerol Oxidation Performance with Highly-Dispersed Pt Nanoclusters Loaded on 3d Graphene-Like Microporous Carbon. Appl. Catal. B Environ..

[41] Huang L., Sun J.-Y., Cao S.-H., Zhan M., Ni Z.-R., Sun H.-J., Chen Z., Zhou Z.-Y., Sorte E.G., Tong Y.J. (2016). Combined EC-NMR and In Situ FTIR Spectroscopic Studies of Glycerol Electrooxidation on Pt/C, PtRu/C, and PtRh/C. ACS Catal..

[42] Cassani A., Tuleushova N., Wang Q., Guesmi H., Bonniol V., Cambedouzou J., Tingry S., Bechelany M., Cornu D., Holade Y. (2021). Fe-Modified Pd as an Effective Multifunctional Electrocatalyst for Catalytic Oxygen Reduction and Glycerol Oxidation Reactions in Alkaline Media. ACS Appl. Energy Mater..

[43] Wang H., Thia§ L., Li N., Ge X., Liu Z., Wang X. (2015). Pd Nanoparticles on Carbon Nitride–Graphene for the Selective Electro-Oxidation of Glycerol in Alkaline Solution. ACS Catal..

[44] Kim D., Lim W.-G., Kim Y., Oh L.S., Kim S., Park J.H., Jo C., Kim H.J., Kang J., Lee S. (2023). Amorphous Antimony Oxide as Reaction Pathway Modulator toward Electrocatalytic Glycerol Oxidation for Selective Dihydroxyacetone Production. Appl. Catal. B Environ..

[45] Lan B., Huang M., Wei R.-L., Wang C.-N., Wang Q.-L., Yang Y.-Y. (2020). Ethanol Electrooxidation on Rhodium–Lead Catalysts in Alkaline Media: High Mass Activity, Long-Term Durability, and Considerable CO_2_ Selectivity. Small.

[46] Zhang M., Xu Z.-H., Liu B.-Y., Duan Y., Zheng Z.-K., Li L.-J., Zhou Q., Matveeva V.G., Hu Z.-F., Yu J. (2023). Anchoring Hydroxyl Intermediate on NiCo(OOH)_X_ Nanosheets to Enable Highly Efficient Electrooxidation of Benzyl Alcohols. AlChE J..

[47] Duan Y., Xue M.-F., Liu B., Zhang M., Wang Y.-C., Wang B.-J., Zhang R.-G., Yan K. (2024). Integration of Theory Prediction and Experimental Electrooxidation of Glycerol On NiCo_2_O_4_ Nanosheets. Chin. J. Catal..

[48] Liu Y., Lan B., Yang Y. (2022). Boosting Ethanol Electrooxidation at RhBi Alloy and Bi_2_O_3_ Composite Surfaces in Alkaline Media. J. Mater. Chem. A.

[49] Huang Z., Hu S., Sun M., Xu Y., Liu S., Ren R., Zhuang L., Chan T.-S., Hu Z., Ding T. (2024). Implanting Oxophilic Metal in PtRu Nanowires for Hydrogen Oxidation Catalysis. Nat. Commun..

[50] Wu J.-C., Kong Z.-J., Li Y.-Y., Lu Y.-X., Zhou P., Wang H.-F., Xu L.-T., Wang S.-Y., Zou Y.-Q. (2022). Unveiling the Adsorption Behavior and Redox Properties of PtNi Nanowire for Biomass-Derived Molecules Electrooxidation. ACS Nano.

[51] Lebedeva N.P., Koper M.T.M., Feliu J.M., van Santen R.A. (2002). Role of Crystalline Defects in Electrocatalysis:  Mechanism and Kinetics of CO Adlayer Oxidation on Stepped Platinum Electrodes. J. Phys. Chem. B.

[52] Lebedeva N.P., Koper M.T.M., Feliu J.M., van Santen R.A. (2002). Mechanism and Kinetics of the Electrochemical CO Adlayer Oxidation on Pt(111). J. Electroanal. Chem..

[53] Bergelin M., Herrero E., Feliu J.M., Wasberg M. (1999). Oxidation of CO Adlayers on Pt(111) at low potentials: An Impinging jet Study in H_2_SO_4_ Electrolyte with Mathematical Modeling of the Current Transients. J. Electroanal. Chem..

[54] Eiler K., Suriñach S., Sort J., Pellicer E. (2020). Mesoporous Ni-Rich Ni–Pt Thin Films: Electrodeposition, Characterization and Performance toward Hydrogen Evolution Reaction in Acidic Media. Appl. Catal. B Environ..

[55] Wang J.-Y., Zhang H.-X., Jiang K., Cai W.-B. (2011). From HCOOH to CO at Pd Electrodes: A Surface-Enhanced Infrared Spectroscopy Study. J. Am. Chem. Soc..

[56] Zhu C., Lan B., Wei R.-L., Wang C.-N., Yang Y.-Y. (2019). Potential-Dependent Selectivity of Ethanol Complete Oxidation on Rh Electrode in Alkaline Media: A Synergistic Study of Electrochemical ATR-SEIRAS and IRAS. ACS Catal..

[57] Yang Y.-Y., Zhang H.-X., Cai W.-B. (2013). Recent Experimental Progresses on Electrochemical ATR-SEIRAS. J. Electrochem..

[58] Wei R.-L., Liu Y., Ma H.-Z., Ma X.-Y., Yang Y.-Y. (2023). Effective Ethanol-to-CO_2_ Electrocatalysis at Iridium-Bismuth Oxide Featuring the Impressive Negative Shifting of the Working Potential. J. Energy Chem..

[59] Ma X.-Y., Ma H.-Z., He S.-H., Zhang Y., Yi Y.-N., Yang Y.-Y. (2023). The Electrocatalytic Activity and Selectivity of Ethylene Glycol Oxidation into Value-Added Chemicals at Iron-Group Electrodes in Alkaline Media. Mater. Today Phys..

