# Supplementary material for: Improving the Electrochemical Glycerol-to-Glycerate Conversion at Pd Sites via the Interfacial Hydroxyl Immigrated from Ni Sites
Source: Molecules. 2024 Aug 16;29(16):3890. doi: 10.3390/molecules29163890 (PMC11356846; doi:10.3390/molecules29163890)
Supplement: Supplementary file 1 [file molecules-29-03890-s001.zip › molecules-3137064-supplementary.pdf]

Supplementary Information

Improving the Electrochemical Glycerol-to-Glycerate Conversion at Pd Sites via the Interfacial Hydroxyl Immigrated from Ni Sites

Yang Zhang [1], Lin Wang [1], Shengmin Pan [1], Lin Zhou [1], Man Zhang [1,*], Yaoyue Yang [1,*] and Wenbin Cai [2,*]

[1] Key Laboratory of General Chemistry of the National Ethnic Affairs Commission, School of Chemistry and Environment, Southwest Minzu University, Chengdu 610041, China; 220703042004@stu.swun.edu.cn (Y.Z.); wanglin1320310@163.com (L.W.); 202231201091@stu.swun.edu.cn (S.P.); 202231201174@stu.swun.edu.cn (L.Z.)

[2] Shanghai Key Laboratory of Molecular Catalysis and Innovative Materials, Collaborative Innovation Center of Chemistry for Energy Materials, Department of Chemistry, Fudan University, Shanghai 200438, China

* Correspondence: zhangm289@swun.edu.cn (M.Z.); yaoyueyoung@swun.edu.cn (Y.Y.); wbcai@fudan.edu.cn (W.C.)

Contents

1. Figures and Tables

2. References

Figures and Tables

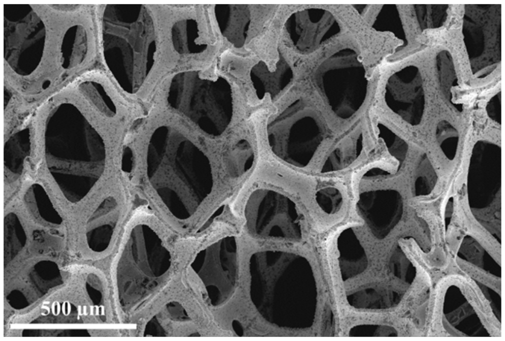

Figure S1. SEM image of Pd/NF.

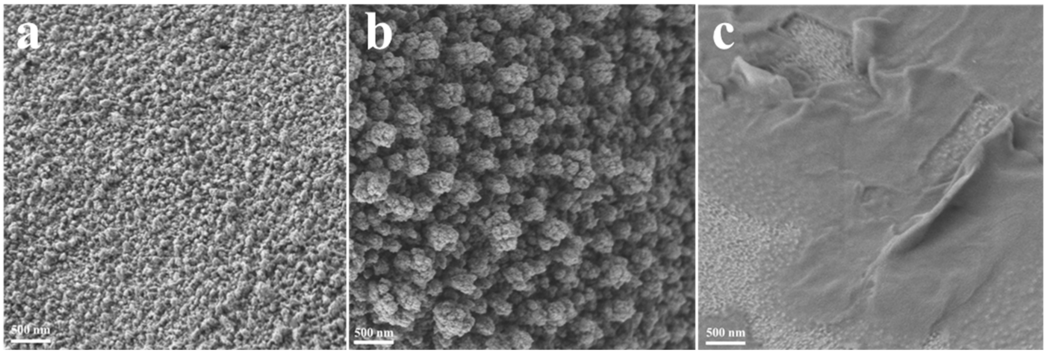

Figure S2. SEM images of (a) Pd/NF at the deposition potential of -0.23 V vs. RHE, (b) Pd/NF at the deposition potential of -0.03 V vs. RHE, (c) Pd/NF at the deposition potential of 0.17 V vs. RHE.

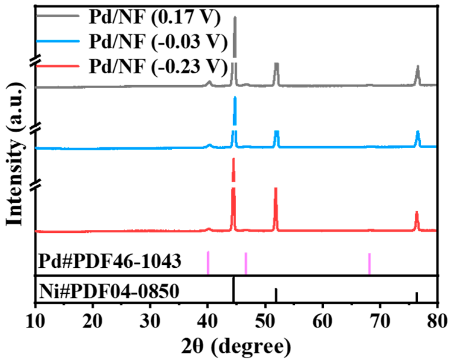

Figure S3. XRD patterns of Pd/NF catalyst (at different deposition potentials of -0.23, -0.03, and 0.17 V vs. RHE).

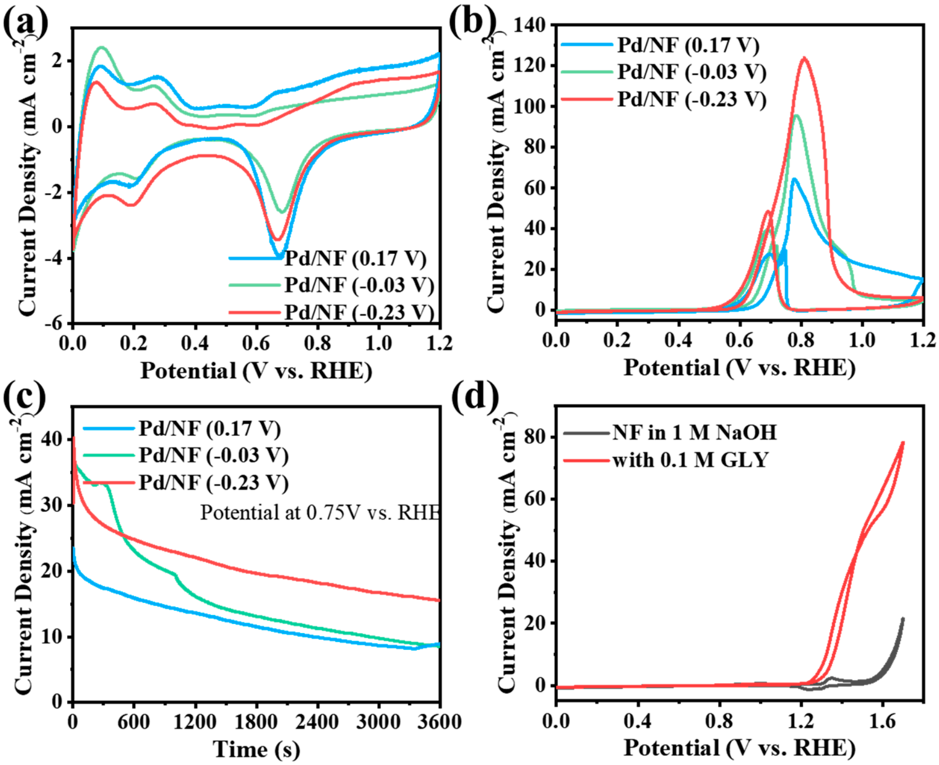

Figure S4. The performance of Pd/NF at different deposition potentials: (a) Cyclic voltammetry in 1 M NaOH electrolyte. (b) Cyclic voltammetry in 1 M NaOH and 0.1 M glycerol electrolyte. (c) *i*-t curves were taken at 0.75 V vs. RHE. (d) Cyclic voltammetry at NF electrode in 1 M NaOH without and with 0.1 M glycerol.

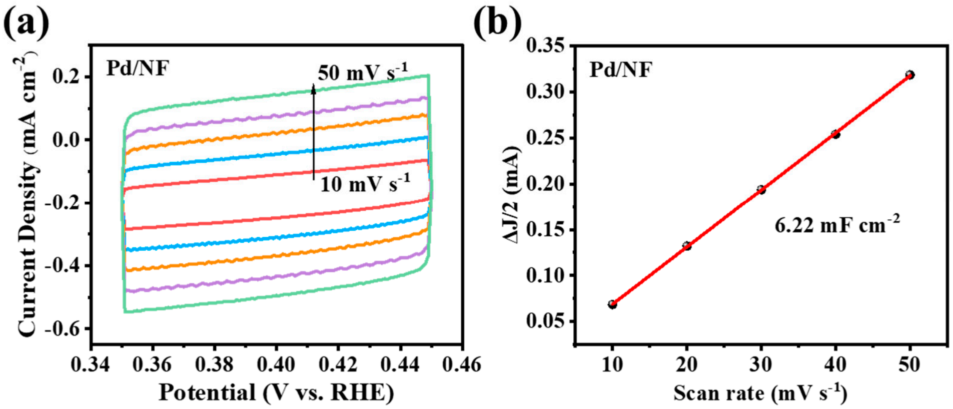

Figure S5. Electrochemical surface area (ECSA) tests at Pd/NF in 1 M NaOH and 0.1 M glycerol electrolyte. (a) Electrochemical cyclic voltammetry on Pd/NF at the scan

rate of 10, 20, 30, 40, and 50 mV s^{-1}. (b) Linear fittings of the capacitive currents versus scan rate at Pd/NF.

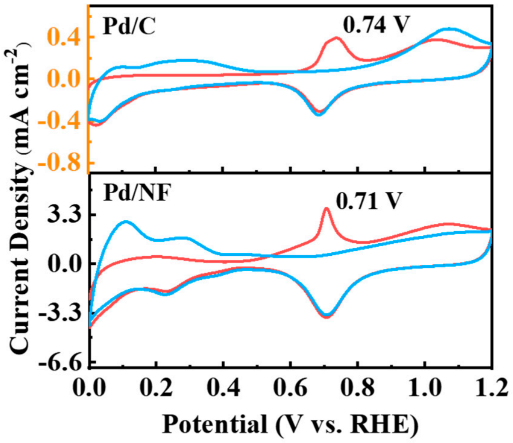

Figure S6. CO stripping curves of Pd/C and Pd/NF in 1 M NaOH without and with 0.1 M glycerol.

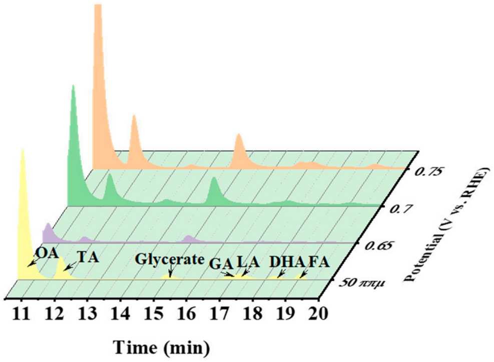

Figure S7. The high-performance liquid chromatography (HPLC) of products over Pd/NF catalyst during the electrocatalytic glycerol oxidation reaction (GOR) for 1-hour in 1 M NaOH and 0.1 M glycerol at different potential.

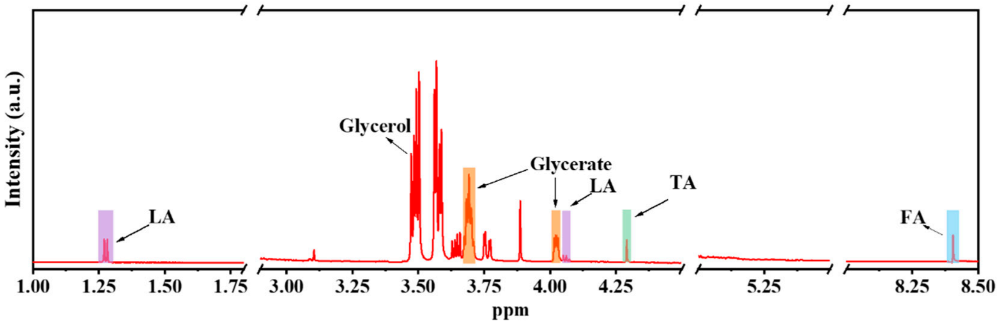

Figure S8. ^{1}H NMR spectra of glycerol electrooxidation products over Pd/NF. The peak at 1.27 and 4.06 ppm assigned to LA, and 3.49 ppm versus glycerol, 3.69 and 4.02 ppm versus glycerate, 4.29 ppm versus TA, 8.4 ppm versus FA [1].

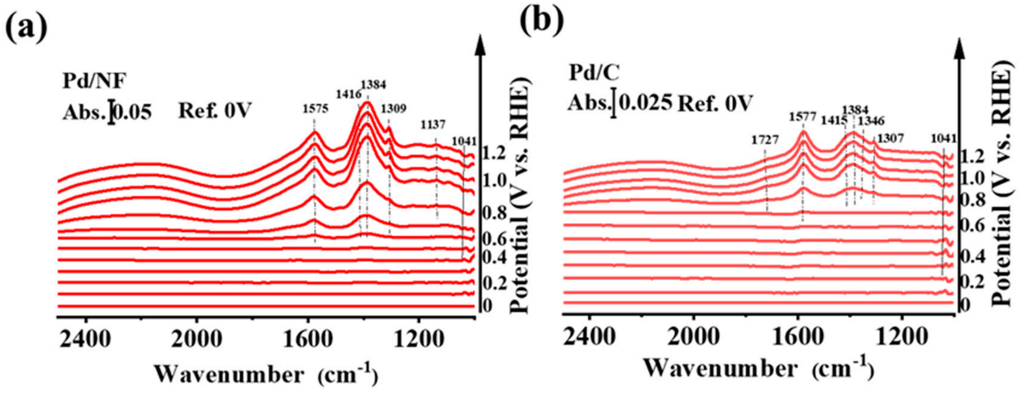

Figure S9. *In-situ* IRAS spectra collected on (a) Pd/NF and (b) Pd/C in 1 M NaOH and 0.1 M glycerol electrolyte at a potential scanning rate of 5 mV s^{-1}, spectral resolution of 8 cm^{-1}.

The downward absorption peak at 1041 cm^{-1} can be attributed to the OH vibration of the alcohol molecule, indicating the consumption of glycerol reactants [2, 3]. Two upward absorption bands near 1416 (1415) and 1575 (1577) cm^{-1}, belonging

to symmetric and asymmetric O-C-O stretching, demonstrate the formation of carboxylate ions, including glycerate, tartrate, and lactate ions, respectively, during the reactions on the two catalysts. The peak near 1384 cm^{-1} may correspond to the presence of CO_3^{2-} [2]. And the Pd/NF electrocatalytic GOR has a faster reaction rate as can be seen from the peak intensities in the figure.

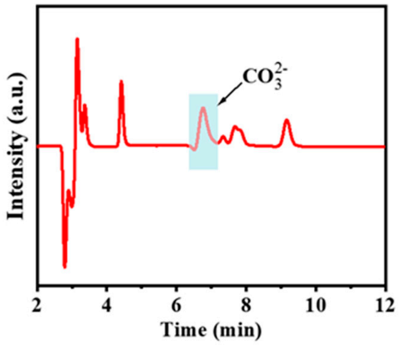

Figure S10. Ion chromatography of electrolyte after 6-hour electrolysis at 0.75 V vs. RHE.

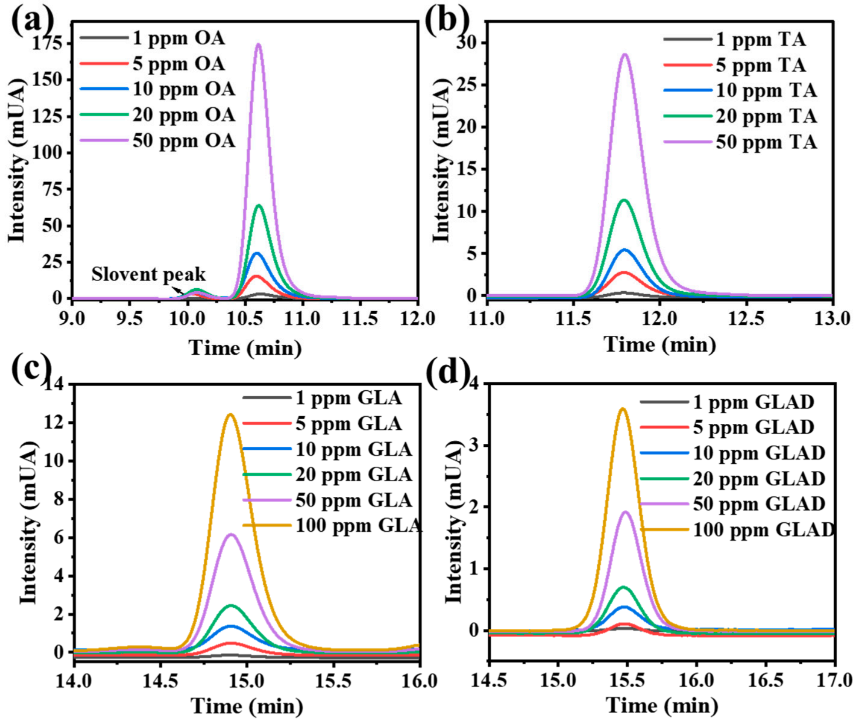

Figure S11. HPLC of (a) oxalic acid (OA), (b) tartaric acid (TA), (c) glyceric acid (GLA), and (d) glyceraldehyde (GLAD).

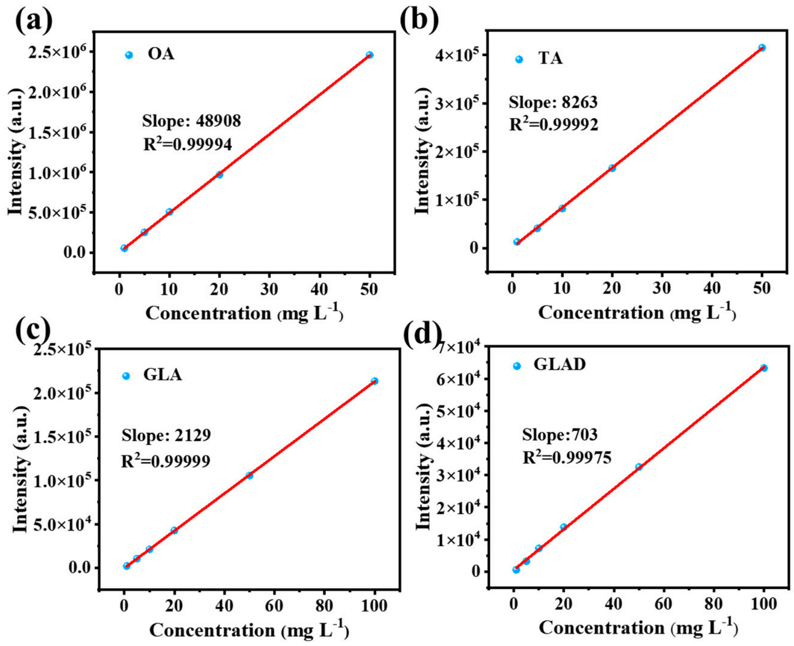

Figure S12. The HPLC calibration curves of (a) oxalic acid (OA), (b) tartaric acid (TA), (c) glyceric acid (GLA), and (d) glyceraldehyde (GLAD).

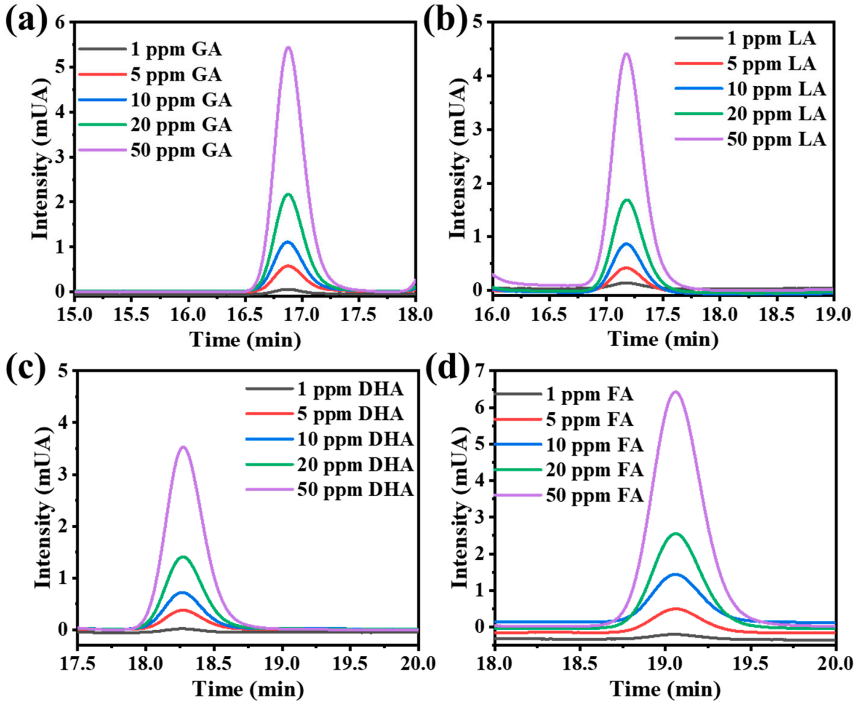

Figure S13. HPLC of (a) glycolic acid (GA), (b) lactic acid (LA), (c) dihydroxyacetone (DHA), and (d) formic acid (FA).

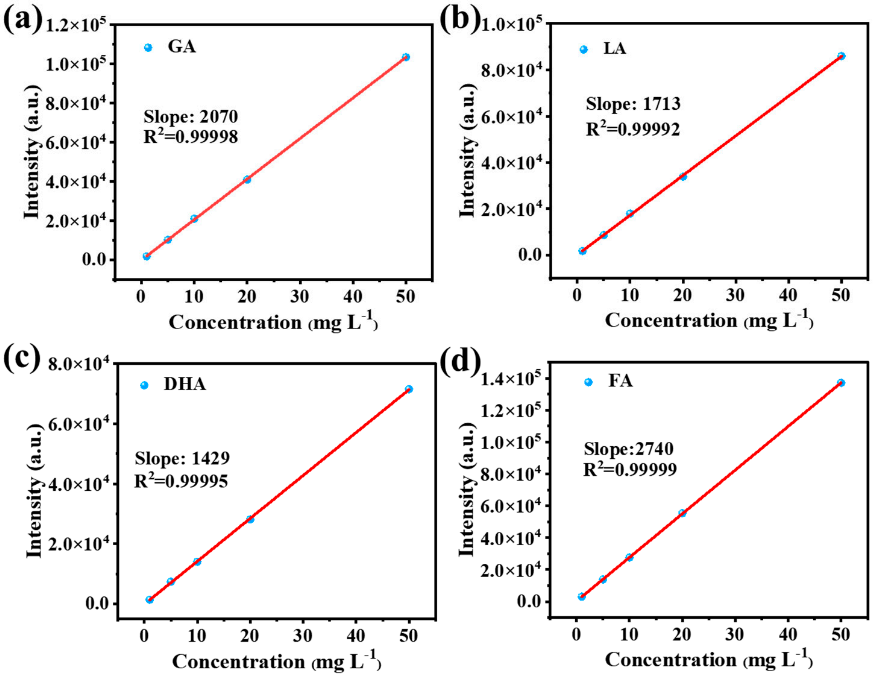

Figure S14. The HPLC calibration curves of (a) glycolic acid (GA), (b) lactic acid (LA), (c) 1-3 dihydroxyacetone (DHA), and (d) formic acid (FA).

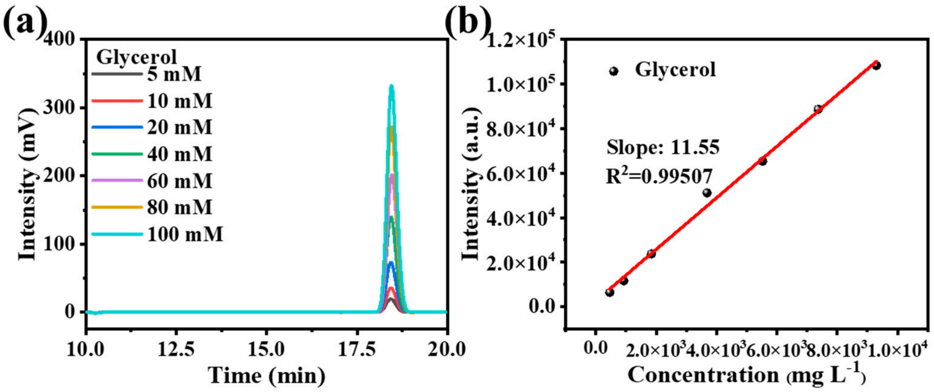

Figure S15. (a) The quantitative determination of glycerol detected by differential refractive index detector. The flow rate of the eluent (0.5 mM H_2SO_4) was 0.4 mL min^{-1}. 20 µL of electrolyte (electrolyte was diluted by 2 times with 0.5 M H_2SO_4) was inject into the column under the temperature of the column at 50 ℃. (b) The HPLC calibration curve of glycerol.

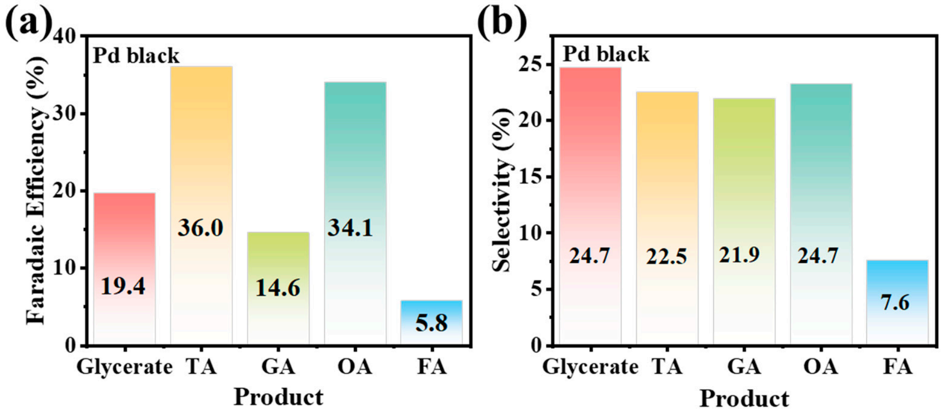

Figure S16. (a) *FE* and (b) selectivity at Pd black catalyst (at the potential of 0.75 V vs. RHE) in 1 M NaOH with 0.1 M glycerol after 1-hour electrolysis.

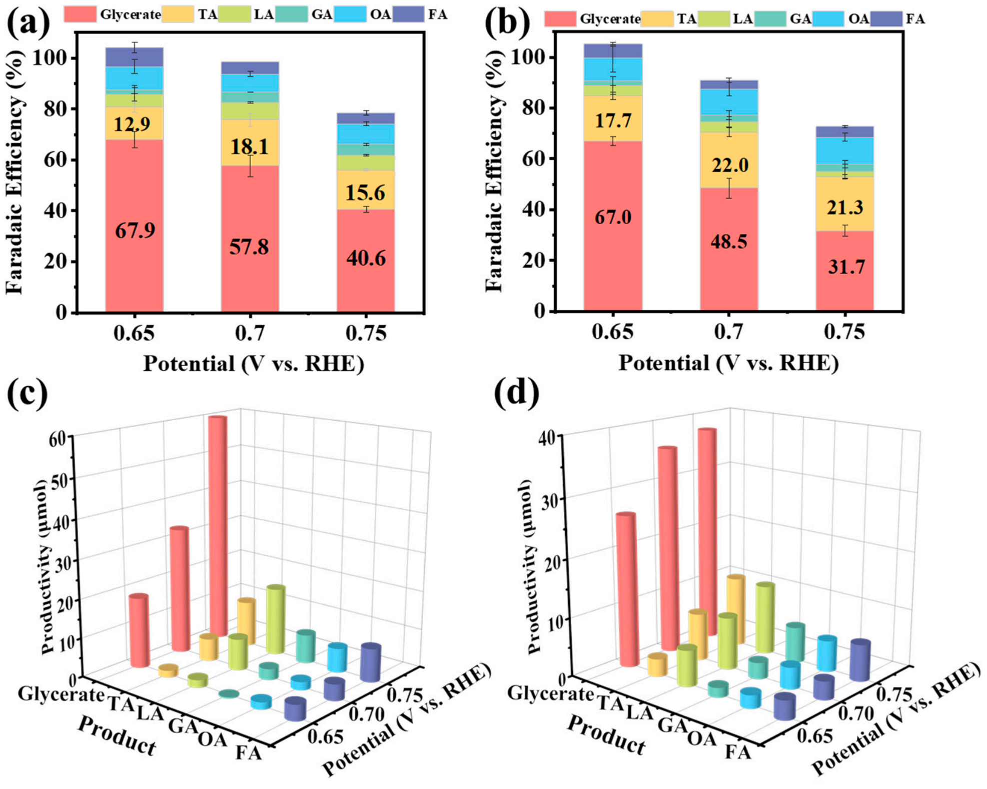

Figure S17. The *FE* of glycerol at (a) Pd/NF (-0.03 V vs. RHE) and (b) Pd/NF (0.17 V vs. RHE) in 1 M NaOH with 0.1 M glycerol after 1-hour electrolysis. Productivity of (c) Pd/NF (-0.03 V vs. RHE) and (d) Pd/NF (0.17 V vs. RHE) in 1 M NaOH with 0.1 M glycerol after 1-hour electrolysis.

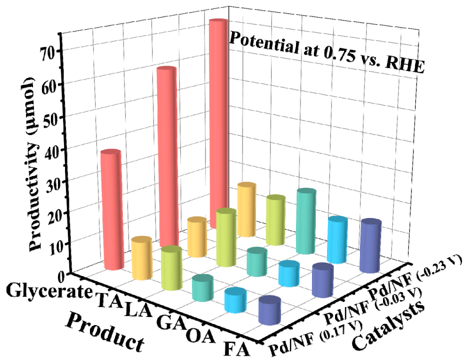

Figure S18. Productivity of Pd/NF with different deposition potentials in 1 M NaOH with 0.1 M glycerol after 1-hour electrolysis.

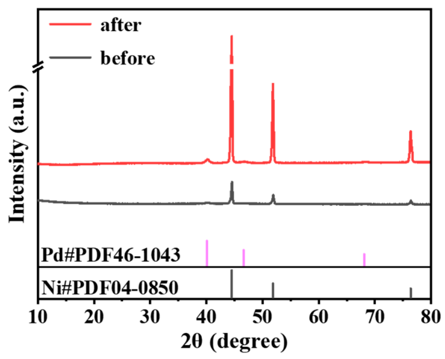

Figure S19. XRD patterns of the Pd/NF catalyst before and after GOR in 1 M NaOH and 0.1 M glycerol electrolyte.

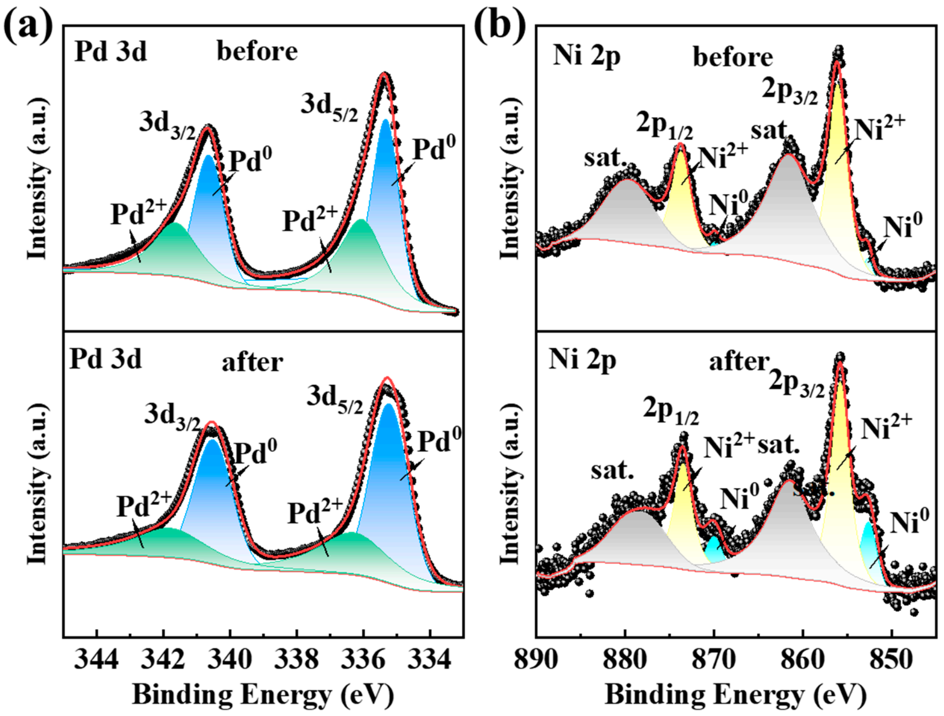

Figure S20. (a) Pd 3d XSP patterns of the Pd/NF catalyst before and after electrocatalytic GOR. (b) Ni 2p XSP patterns of the Pd/NF catalyst before and after electrocatalytic GOR.

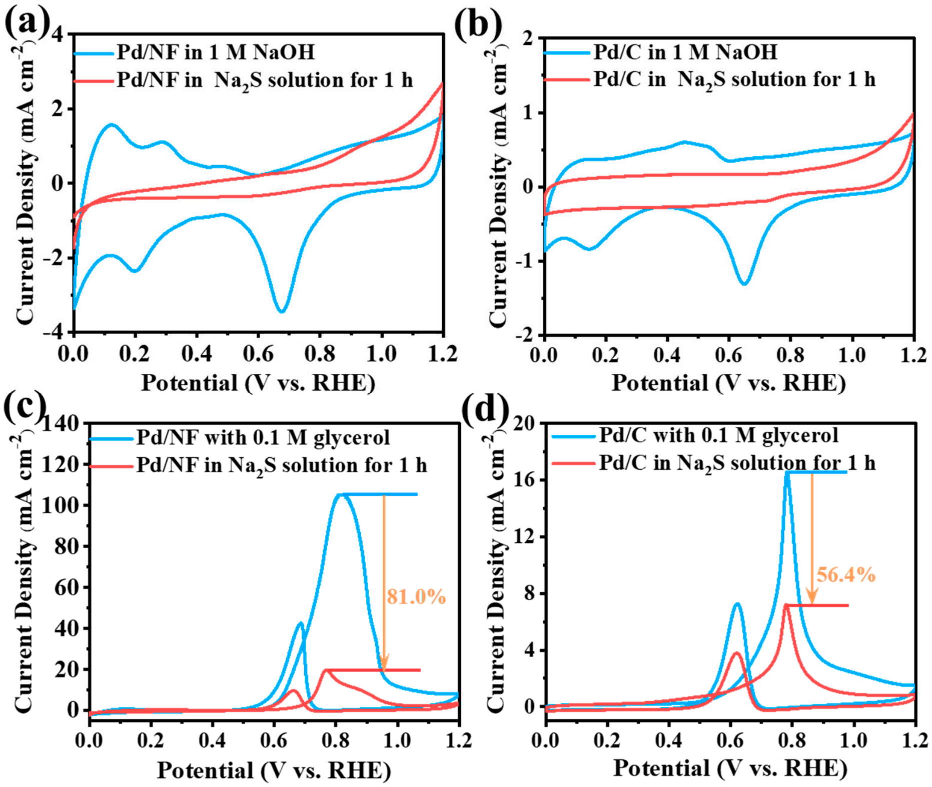

Figure S21. (a) Cyclic voltammetry of Pd/NF (b) Cyclic voltammetry of Pd/C and (with or without immersing in 3.8 mM Na_2S solution for 1 h) in 1 M NaOH electrolyte. (c) Cyclic voltammetry of Pd/NF and (d) Cyclic voltammetry of Pd/C (with or without immersing in 3.8 mM Na_2S solution for 1 hour) in 1 M NaOH and 0.1 M glycerol electrolyte.

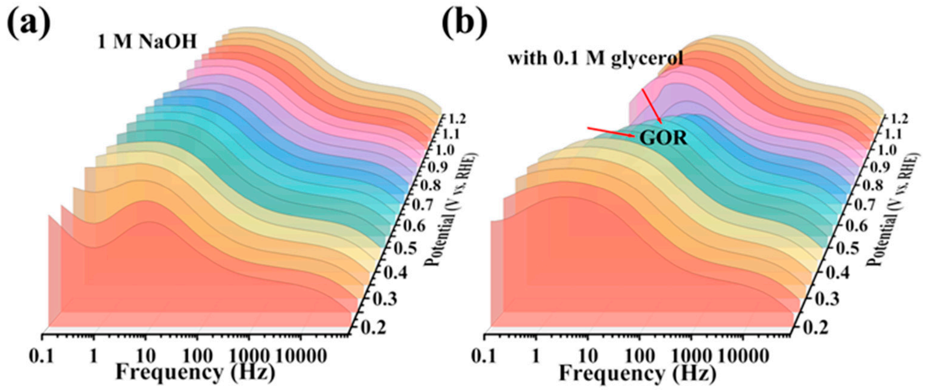

Figure S22. Bode plots of Pd/NF in 1 M NaOH solution without (a) and with (b) 0.1 M glycerol under different potentials using *operando* EIS analysis.

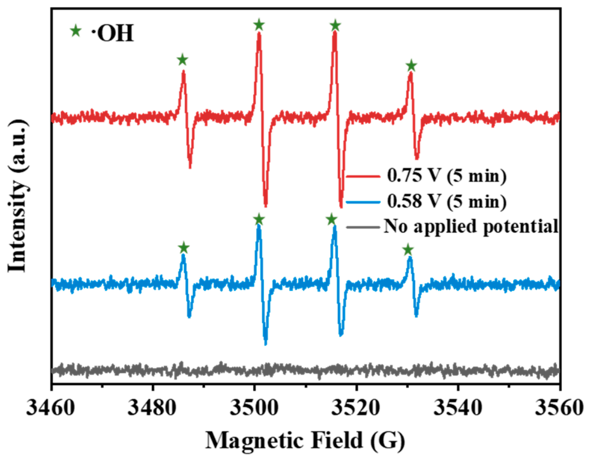

Figure S23. EPR spectra after the Pd/NF working electrode were applied potential of 0.58 and 0.75 V vs. RHE in 1 M NaOH with 0.1 M glycerol for 300 s using DMPO as the trapping agent

Table S1 Loading mass of Pd on Ni foam for Pd/NF catalysts by inductively coupled plasma optical emission spectrometer (ICP-OES).

**Catalysts**	**Pd mass loading on Ni foam (wt%)**	**Pd load capacity** (mg cm^{-2})
Pd/NF (0.17 V)	1.25	0.82
Pd/NF (-0.03 V)	1.60	1.05
Pd/NF (-0.23 V)	3.10	2.04

Table S2. Comparison of the C3 chemicals for GOR in the reported work and in this work.

**Catalysts**	**Electrolyte(condition)**	**FE of C_3 chemicals (%)**	**Selectivity of C_3 chemicals (%)**	**Ref.***
PdNi/C<br>PdAg/C	0.1 M NaOH + 0.1 M glycerol(2h)<br>0.8 V_{RHE}	/	GLA (~39)<br>GLA (~34)	[4]
Pd-CN_x/G	0.5 M NaOH+0.5 M glycerol<br>0.95 V_{RHE}	/	**GLA** (~27.6)<br>TA (0.7)	[5]
PdFe/rGO	1 M NaOH + 0.1 M glycerol (2h)<br>0.8 V_{RHE}	/	GLA (~17)<br>TA (6)	[6]
LANP Pd	1 M NaOH + 0.1 M glycerol	GLAD (3.4)	GLA (~56)	[7]

	0.8 V_{RHE}	GLA (44.38) TA (17.03)		
LANP PdCu7525		GLAD (15.49) GLA (40.10) TA (16.37)	GLAD (81) GLA (15) TA (3)	
LANP PdCu5050		GLAD (67.17) GLA (20.85) TA (8.75)	GLA (~40)	
Pd-CFP (OCTA)	1 M NaOH + 0.1 M glycerol (0.5 h) 60 °C 0.86 V_{RHE}	/	GLA (50) LA (11) TA (10)	[8]
Pt/FeNC	8.0 M KOH +5.0 M glycerol (20 h) 80 °C 0.9 V_{RHE}		GLA (28.3) TA (55.5) LA (6.3)	[9]
Pt(1%)Ag/C	0.5 M NaOH + 1 M glycerol (0.5 h) 0.8 V_{RHE}		GLA (36.97) LA (26.74)	[10]
Pt/3D-GLC<br>Pt/C	0.5 M H_2SO_4+2 M glycerol (6 h) 60 °C 1.136 V_{SHE}	/	GLA (42.7) GALD (56)<br>GALD (99.2)	[11]
MoOx/Pt	1 M KOH+0.1 M glycerol (1 h) 0.8 V_{RHE}	/	GLA (73)	[12]

Pt_3Ru_1/C	0.1 M $HClO_4$+0.1 M glycerol (8 h) 60 °C 0.63 V_{RHE}	/	GLA (~40)	[13]
planar Au<br>RA-Au	1 M KOH+ 0.1 M glycerol<br>0.9 V_{RHE} (4 h)	GLA (43.76)<br>LA (20.03)<br>GLA (18.34)<br>LA (13.64)	GLA (32.56)<br>LA (29.45)<br>GLA (11.78)<br>LA (17.88)	[14]
$PtNi_2$	1 M KOH +0.1 M glycerol (1 h)<br>0.5 V_{RHE}	GLA (52) TA (48)	/	[15]
Pt/C	1 M KOH + 0.5 M glycerol	GLA (~25)	/	[16]
Pd/NF	1 M NaOH + 0.1 M glycerol (1 h) 25°C 0.65 V_{RHE}	GLA (65.6) TA (17) LA (3.17)	GLA (70.5) TA (8.6) LA (6.8)	This work

References

1. Li, Y.; Wei, X.; Pan, R.; Wang, Y.; Luo, J.; Li, L.; Chen, L.; Shi, J., PtAu Alloying-Modulated Hydroxyl and Substrate Adsorption for Glycerol Electrooxidation to C_3 Products. *Energy Environ. Sci.* **2024,** 17, (12), 4205-4215.
2. Huang, B.; Ge, Y.; Zhang, A.; Zhu, S.; Chen, B.; Li, G.; Yun, Q.; Huang, Z.; Shi, Z.; Zhou, X.; Li, L.; Wang, X.; Wang, G.; Guan, Z.; Zhai, L.; Luo, Q.; Li, Z.; Lu, S.; Chen, Y.; Lee, C.-S.; Han, Y.; Shao, M.; Zhang, H., Seeded Synthesis of Hollow PdSn Intermetallic Nanomaterials for Highly Efficient Electrocatalytic Glycerol Oxidation. *Adv. Mater.* **2023,** 35, (35), 2302233.
3. Zalineeva, A.; Serov, A.; Padilla, M.; Martinez, U.; Artyushkova, K.; Baranton, S.; Coutanceau, C.; Atanassov, P. B., Glycerol Electrooxidation on Self-Supported Pd_1Sn_x Nanoparticules. *Appl. Catal. B Environ.* **2015,** 176-177, 429.
4. Holade, Y.; Morais, C.; Servat, K.; Napporn, T. W.; Kokoh, K. B., Toward the Electrochemical Valorization of Glycerol: Fourier Transform Infrared Spectroscopic and Chromatographic Studies. *ACS Catal.* **2013,** 3, (10), 2403-2411.
5. Wang, H.; Thia§, L.; Li, N.; Ge, X.; Liu, Z.; Wang, X., Pd Nanoparticles on Carbon Nitride–Graphene for the Selective Electro-Oxidation of Glycerol in Alkaline Solution. *ACS Catal.* **2015,** 5, (6), 3174-3180.

6. Cassani, A.; Tuleushova, N.; Wang, Q.; Guesmi, H.; Bonniol, V.; Cambedouzou, J.; Tingry, S.; Bechelany, M.; Cornu, D.; Holade, Y., Fe-Modified Pd as an Effective Multifunctional Electrocatalyst for Catalytic Oxygen Reduction and Glycerol Oxidation Reactions in Alkaline Media. *ACS Appl. Energy Mater.* **2021,** 4, (9), 9944-9960.
7. Mo, X.; Gao, X.; Gillado, A. V.; Chen, H.-Y.; Chen, Y.; Guo, Z.; Wu, H.-L.; Tse, E. C. M., Direct 3D Printing of Binder-Free Bimetallic Nanomaterials as Integrated Electrodes for Glycerol Oxidation with High Selectivity for Valuable C_3 Products. *ACS Nano* **2022,** 16, (8), 12202-12213.
8. Terekhina, I.; White, J.; Cornell, A.; Johnsson, M., Electrocatalytic Oxidation of Glycerol to Value-Added Compounds on Pd Nanocrystals. *ACS Appl. Nano Mater.* **2023,** 6, (13), 11211-11220.
9. Li, J.; Jiang, K.; Bai, S.; Guan, C.; Wei, H.; Chu, H., High Productivity of Tartronate from Electrocatalytic Oxidation of High Concentration Glycerol through Facilitating the Intermediate Conversion. *Appl. Catal. B Environ.* **2022,** 317, 121784.
10. Lima, C. C.; Rodrigues, M. V. F.; Neto, A. F. M.; Zanata, C. R.; Pires, C. T. G. V. M. T.; Costa, L. S.; Solla-Gullón, J.; Fernández, P. S., Highly Active Ag/C Nanoparticles Containing Ultra-Low Quantities of Sub-Surface Pt for the Electrooxidation of Glycerol in Alkaline Media. *Appl. Catal. B Environ.* **2020,** 279, 119369.
11. Lee, D.; Kim, Y.; Kwon, Y.; Lee, J.; Kim, T.-W.; Noh, Y.; Kim, W. B.; Seo, M. H.; Kim, K.; Kim, H. J., Boosting the Electrocatalytic Glycerol Oxidation Performance with Highly-Dispersed Pt Nanoclusters Loaded on 3d Graphene-Like Microporous Carbon. *Appl. Catal. B Environ.* **2019,** 245, 555.
12. Yu, X.; dos Santos, E. C.; White, J.; Salazar-Alvarez, G.; Pettersson, L. G. M.; Cornell, A.; Johnsson, M., Electrocatalytic Glycerol Oxidation with Concurrent Hydrogen Evolution Utilizing an Efficient MoO_x/Pt Catalyst. *Small* **2021,** 17, (44), 2104288.
13. Huang, L.; Sun, J.-Y.; Cao, S.-H.; Zhan, M.; Ni, Z.-R.; Sun, H.-J.; Chen, Z.; Zhou, Z.-Y.; Sorte, E. G.; Tong, Y. J.; Sun, S.-G., Combined EC-NMR and In Situ FTIR Spectroscopic Studies of Glycerol Electrooxidation on Pt/C, PtRu/C, and PtRh/C. *ACS Catal.* **2016,** 6, (11), 7686-7695.
14. Kim, D.; Oh, L. S.; Tan, Y. C.; Song, H.; Kim, H. J.; Oh, J., Enhancing Glycerol Conversion and Selectivity toward Glycolic Acid via Precise Nanostructuring of Electrocatalysts. *ACS Catal.* **2021,** 11, (24), 14926-14931.
15. Luo, H.; Yukuhiro, V. Y.; Fernández, P. S.; Feng, J.; Thompson, P.; Rao, R. R.; Cai, R.; Favero, S.; Haigh, S. J.; Durrant, J. R.; Stephens, I. E. L.; Titirici, M.-M., Role of Ni in PtNi Bimetallic Electrocatalysts for Hydrogen and Value-Added Chemicals Coproduction via Glycerol Electrooxidation. *ACS Catal.* **2022,** 12, (23), 14492-14506.
16. Yadegari, H.; Ozden, A.; Alkayyali, T.; Soni, V.; Thevenon, A.; Rosas-Hernández, A.; Agapie, T.; Peters, J. C.; Sargent, E. H.; Sinton, D., Glycerol Oxidation Pairs with Carbon Monoxide Reduction for Low-Voltage Generation of C_2 and C_3 Product Streams. *ACS Energy Lett.* **2021,** 6, (10), 3538-3544.
